# Mental health, financial, and social outcomes among older adults with probable COVID-19 infection: A longitudinal cohort study

**DOI:** 10.1073/pnas.2200816119

**Published:** 2022-06-28

**Authors:** Eleonora Iob, Andrew Steptoe, Paola Zaninotto

**Affiliations:** ^a^Department of Behavioural Science and Health, University College London, WC1E 7HB, London, United Kingdom;; ^b^Department of Epidemiology and Public Health, University College London, WC1E 7HB, London, United Kingdom

**Keywords:** COVID-19 infection, mental health, financial hardship, social connections, older adults

## Abstract

We investigated the immediate and longer-term impact (over 4-6 months) of probable COVID-19 infection on mental health, wellbeing, financial hardship, and social interactions among older people living in England. Data were analysed from 5146 older adults participating in the English Longitudinal Study of Ageing who provided data before the pandemic (2018-19) and at two COVID-19 assessments in 2020 (June-July and November-December). The associations of probable COVID-19 infection (first COVID-19 assessment) with depression, anxiety, poor quality of life (QoL), loneliness, financial hardship, and social contact with family/friends at the first and second COVID-19 assessments were tested using linear/logistic regression and were adjusted for pre-pandemic outcome measures. Participants with probable infection had higher levels of depression and anxiety, poorer QoL, and greater loneliness scores compared with those without probable infection at both the first (OR_depression_ = 1.62, *P*-value = 0.005; OR_anxiety_ = 1.59, *P*-value = 0.049; b_poorQoL_ = 1.34, *P* < 0.001; b_loneliness_ = 0.49, *P* < 0.001) and second (OR_depression_ = 1.56, *P*-value = 0.003; OR_anxiety_ = 1.55, *P*-value = 0.041; b_poorQoL_ = 1.38, *P*-value < 0.001; b_loneliness_ = 0.31, *P*-value = 0.024) COVID-19 assessments. Participants with probable infection also experienced greater financial difficulties than those without infection at the first assessment (OR = 1.50, *P*-value = 0.011). Probable COVID-19 infection is associated with longer-term deterioration of mental health and wellbeing and short-term increases in financial hardship among older adults. It is important to monitor the mental health of older people affected by COVID-19 and provide additional support to those in need.

## Significance

Longitudinal evidence on the impact that contracting COVID-19 may have on the individual’s mental health, personal finances, and social relationships is scarce. Using longitudinal data from the English Longitudinal Study of Aging, this study shows that older adults with probable COVID-19 infection experienced higher levels of depression and anxiety, poorer quality of life, elevated feelings of loneliness, and greater financial difficulties compared with those without probable infection. The associations were independent of prepandemic mental health and financial circumstances, and they were evident both in the acute phase of the infection and up to 6 months later. These results suggest that the adverse psychosocial impact of COVID-19 infection is long-lasting and more broadly present across the population.

The coronavirus disease 2019 (COVID-19) pandemic has affected several aspects of people’s lives, including physical and mental health, employment and financial security, social connections, and access to healthcare (1). Despite a large body of research documenting the adverse psychosocial effects of the pandemic and its containment measures across the population, little is currently known regarding the impact that contracting COVID-19 itself may have on the individual’s mental health, personal finances, and social relationships.

Several longitudinal studies have reported increases in depression, anxiety, and general psychological distress in the adult population during the COVID-19 pandemic compared with prepandemic levels (2, 3). People who have contracted COVID-19 might be particularly vulnerable to the psychological impact of the pandemic. Indeed, initial evidence suggests that the experience of COVID-19 symptoms is associated not only with adverse physical consequences, but also with long-term effects on mental health (4, 5). Various mechanisms could underlie the psychological effects of COVID-19 infection, including the potential neurotropic properties of the virus (6, 7); the presence of elevated proinflammatory cytokines (e.g., interleukin-6) in patients with severe COVID-19 symptoms (8), which are implicated in the development of psychiatric disorders such as depression (9); and the exposure to prolonged periods of social isolation and physical inactivity in people affected by COVID-19 (10), which in turn can increase mental distress and feelings of loneliness. Compounded by the widespread psychosocial effects of the pandemic across the population, these factors might further exacerbate the risk of mental health problems among individuals recovering from COVID-19 infection.

Data from previous coronavirus epidemics demonstrate the potential psychiatric consequences of the virus in both the acute and postacute phases of the illness (11). Further, studies across different countries have found that individuals reporting COVID-19 symptoms and patients recovering from acute COVID-19 illness exhibit increased levels of anxiety, depression, suicidal ideation, loneliness, and poor quality of life (QoL) compared with healthy people (5, 12–19). Studies using data from electronic health records in the United States have also shown that COVID-19 patients with no previous psychiatric history are at increased risk of first-time diagnosis of psychiatric disorders compared with those affected by other health events (e.g., influenza) or healthy controls (20, 21). However, most studies to date are limited by small, nonrepresentative samples and short follow-up periods, and they lack longitudinal data on the participants’ mental health before COVID-19, as well as data on confounding factors. Since individuals with preexisting mental disorders seem particularly susceptible to COVID-19 infection (20, 22), it is unclear the extent to which reverse causality and confounding bias might contribute to the association between COVID-19 infection and psychological distress. In addition, studies involving electronic health records or clinical samples may not capture individuals with moderate COVID-19 symptoms and those with less severe mental health problems who do not present to health services.

Longitudinal cohort studies are well suited to study the immediate and longer-term psychosocial consequences of COVID-19 infection in the general population, as they include comprehensive information on mental health before the infection and other confounding factors (e.g., sex, age, socioeconomic position). Results from the United Kingdom suggest that people with probable COVID-19 symptoms experience greater psychological distress up to 7 months following the start of the infection (23). In contrast, an online study in the United States found evidence only for short-term psychological effects that diminish as the symptoms subside (24). Notably, these studies have only focused on general psychological distress; therefore, the impact of COVID-19 infection on specific mental health and wellbeing outcomes (e.g., depression, anxiety, loneliness, and QoL) in the general population is unclear.

Numerous studies have also highlighted the financial impact of the pandemic—including job losses, pay cuts, reductions in household income, fluctuations in stock markets and wealth held in risky assets, and widespread financial worries (25–27)—as well as its adverse consequences for various domains of social relationships, including social networks, social support, and social interaction (28, 29). However, these studies relate to the whole population rather than to people with COVID-19 infection. Empirical evidence regarding the impact that COVID-19 infection may have on a person’s financial situation and social relationships is limited. For instance, cross-sectional results suggest that adults who have experienced COVID-19 are more likely to report that their social relationships, work, and household finances have been adversely affected by the pandemic, compared with those who have not had COVID-19 (30). However, this analysis did not account for preexisting trends in social connections and economic outcomes, and it was unable to disentangle short-term versus longer-term psychosocial consequences of the infection.

Older adults are at increased risk of social isolation and serious illness following COVID-19 infection (31), and they also are particularly vulnerable to the effects of chronic stress on the brain (32). A recent analysis of data from the English Longitudinal Study of Aging (ELSA) also demonstrates that the mental health and wellbeing of the older population deteriorated significantly as the pandemic progressed in 2020, compared with prepandemic levels (33). Given these factors, older people might be disproportionally affected by the psychosocial effects of COVID-19 infection. However, little research on COVID-19 has involved older adults who are also often unable to access online surveys (34). In addition, care-seeking behaviors changed considerably in the early stages of the pandemic, with large numbers of older adults with care needs not actively contacting health services and not seeking help (35). Therefore, older adults’ experiences of COVID-19 might be underrepresented in earlier studies.

In the present analysis, we investigated the immediate and longer-term impact (over 4 to 6 months) of probable COVID-19 infection on mental health (i.e., depression and anxiety), wellbeing (i.e., QoL and loneliness), financial hardship, and social interactions in a large, representative sample of older adults from ELSA. In addition, we assessed whether the psychosocial impact of probable COVID-19 infection might vary across different sociodemographic groups. All outcomes were assessed before the pandemic began (i.e., 2018/2019) and on two occasions during the pandemic, which enabled us to test both short-term and longer-term associations. The data were collected online and by telephone interview to ensure coverage of those without internet access.

## Results

### Descriptive Statistics.

The sample included 5,146 core ELSA members who participated in both COVID-19 assessments (i.e., June to July and November to December 2020) and in the most recent regular ELSA wave before the pandemic (i.e., wave 9, 2018/2019). The number of probable COVID-19 cases at the first COVID-19 assessment (June to July 2020) was 501 (9.7%), according to definition 1 (i.e., a positive COVID-19 test result, hospitalization due to COVID-19, or reporting one of the three core symptoms of COVID-19), which is slightly higher than the estimated prevalence of COVID-19 in England following the first peak of the pandemic (∼6%) (36). The distribution of the covariates and outcomes in participants with and without probable COVID-19 infection at the first COVID-19 assessment is shown in Table 1. Further details are provided in *SI Appendix*, *SI Results*.

**Table 1.** Distribution of the covariates and outcomes in participants with and without probable COVID-19 infection (COVID-19 assessment 1, June to July 2020)

	**Infection: No (*n* = 4,645)**	**Infection: Yes (*n* = 501)**	***P* value**
**Sex**			
Men	47.10%	47.30%	ref
Women	52.90%	52.70%	0.949
**Age group**			
52 to 59	30.60%	43.90%	ref
60 to 74	44.80%	40.50%	0.003
75 and over	24.70%	15.70%	<0.001
**Ethnicity**			
White	93.10%	89.40%	ref
Other	6.90%	10.60%	0.119
**Partnership**			
Partnered	75.70%	72.80%	ref
Nonpartnered	24.30%	27.20%	0.332
**Living alone**			
No	75.70%	72.80%	ref
Yes	24.30%	27.20%	0.326
**Employment status**			
Employed	37.70%	50.40%	ref
Retired	52.90%	37.60%	<0.001
Other not working*	9.30%	12.00%	0.872
**Wealth (tertiles)**			
Low	41.80%	49.00%	ref
Medium	30.20%	27.50%	0.108
High	28.00%	23.50%	0.033
**Limiting longstanding Illness**			
No	49.00%	43.30%	ref
Yes	51.00%	56.70%	0.094
**Vulnerable to COVID-19**			
No	83.40%	84.00%	ref
Yes	16.60%	16.00%	0.821
**Elevated depressive symptoms (CESD-8 ≥ 4)**			
No	78.50%	65.20%	ref
Yes	21.50%	34.80%	<0.001
**Anxiety (GAD-7 ≥ 10)**			
No	91.30%	84.40%	ref
Yes	8.70%	15.60%	0.001
**Poor QoL (CASP-12)**			
Mean (SD)	22.263 (6.348)	24.995 (7.552)	<0.001
Range	12.000 to 46.000	12.000 to 47.000	
**Loneliness**			
Mean (SD)	5.577 (2.011)	6.307 (2.410)	<0.001
Range	2.000 to 12.000	4.000 to 12.000	
**Financial worries**			
No	73.40%	58.70%	ref
Yes	26.60%	41.30%	<0.001
**Current financial situation worse than pre-COVID-19**			
No	80.90%	69.60%	ref
Yes	19.10%	30.40%	<0.001
**Infrequent contact with family**			
No	92.40%	94.60%	ref
Yes	7.60%	5.40%	0.158
**Infrequent contact with friends**			
No	88.10%	90.80%	ref
Yes	11.90%	9.20%	0.139
**Infrequent contact with family and friends (total score)**			
Mean (SD)	23.623 (5.253)	22.911 (5.614)	0.056
Range	8.000 to 32.000	8.000 to 32.000	

Imputed data; percentages and means are estimated using sampling weights; *P* values derived from univariate logistic regression models comparing the distribution of the covariates and outcomes in participants with and without probable COVID-19 infection across the 20 imputed datasets and weighted using survey weights.

*Other not working: 2.4% unemployed; 4.1% permanently sick/disabled; 3.1% looking after family/home.

### Associations of Probable COVID-19 Infection with Mental Health, Financial Hardship, and Social Interactions.

**Fig. 1.**
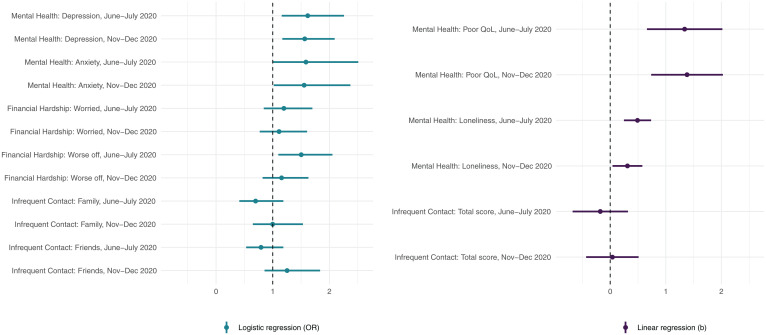
Associations of probable COVID-19 infection (June to July 2020) with mental health, financial hardship, and social connections (95% CIs) at the first and second COVID-19 assessments (June to July 2020 and November to December 2020). ELSA COVID-19 longitudinal sample (n = 5,146); pooled estimates and 95% CIs from logistic/linear regression models across 20 imputed datasets; estimates adjusted for sex, age, pre-COVID-19 outcomes, whether living alone, employment status, wealth, whether vulnerable to COVID-19, November or December 2020 COVID-19 infection (November to December 2020 outcome only), and limiting long-standing illness, and weighted using survey weights.

**Fig. 2.**
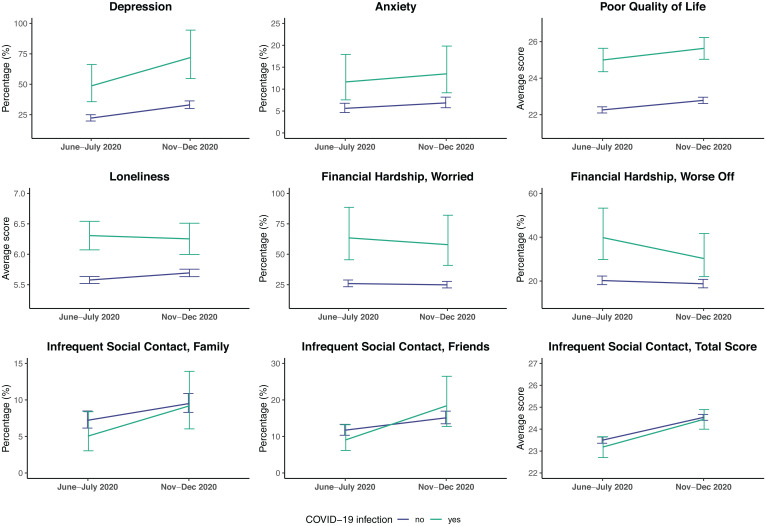
Estimated adjusted percentages and means of the outcomes at the first and second COVID-19 assessments (June to July 2020 and November to December 2020, respectively) in people with and without probable COVID-19 infection (June to July 2020). ELSA COVID-19 longitudinal sample (n = 5,146); pooled estimates and 95% CIs derived from linear/logistic regression models across 20 imputed datasets, adjusted for all covariates and prepandemic outcome scores and weighted using survey weights.

The associations between probable COVID-19 infection and mental health, financial hardship, and social interactions found in the linear/logistic regression analyses are illustrated in Fig. 1 and reported in *SI Appendix*, Table S3. The estimated adjusted percentages and means of the outcomes at the two COVID-19 assessments in people with and without probable COVID-19 infection are shown in Fig. 2. After taking all covariates and prepandemic levels into account, people with probable COVID-19 infection had worse mental health and wellbeing in June and July 2020 than those without probable infection. An estimated 49% (95% CI: 36; 66) of participants with probable COVID-19 infection had clinically significant depressive symptoms, compared with 22% (95% CI: 20; 25) of those without infection (odds ratio [OR] 1.62 [95% CI: 1.16; 2.26] *P* = 0.005); 12% (95% CI: 8; 18) of people with probable infection were identified as having anxiety (cases of generalized anxiety disorder [GAD]), compared with 6% (95% CI: 5; 7) of those without infection (OR 1.59 [95% CI: 1.00; 2.51] *P* = 0.049). Estimated average ratings of poor QoL and loneliness among those with probable infection were 24.99 (95% CI: 24.35; 25.64) and 6.31 (95% CI: 6.07; 6.54), respectively, compared with 22.26 (95% CI: 22.09; 22.43) (b 1.34 [95% CI: 0.66; 2.02] *P* < 0.001) and 5.58 (95% CI: 5.52; 5.63) (b 0.49 [95% CI: 0.25; 0.74] *P* < 0.001) in participants without probable infection.

The adverse impact of probable COVID-19 infection on mental health and wellbeing persisted through to the follow-up assessment in November to December 2020. At this assessment, the estimated prevalence of depression and anxiety among people with probable infection was 72% (95% CI: 55; 94) and 13% (95% CI: 9; 20), respectively, compared with 33% (95% CI: 30; 36) (OR 1.56 [95% CI: 1.17; 2.09] *P* = 0.003) and 7% (95% CI: 6; 8] (OR 1.55 [95% CI: 1.02; 2.37] *P* = 0.041) in those without infection. Further, average ratings of poor QoL and loneliness in people with probable infection were 25.63 (95% CI: 25.03; 26.23) and 6.25 (95% CI: 6.00; 6.51), respectively, compared with 22.78 (95% CI: 22.61; 22.96) (b 1.38 [95% CI: 0.74; 2.03] *P* < 0.001) and 5.69 (95% CI: 5.63; 5.75) (b 0.31 [95% CI: 0.04; 0.58] *P* = 0.024) among those without probable infection. With regard to financial hardship, an estimated 40% (95% CI: 30; 53) of people with probable COVID-19 infection experienced more financial difficulties in June and July 2020 than before the pandemic, compared with 20% (95% CI: 18; 22) of those without infection (OR 1.50 [95% CI: 1.10; 2.05] *P* = 0.011). However, this difference was smaller and nonsignificant in November and December 2020 (OR 1.16 [95% CI: 0.82; 1.63] *P* = 0.407). The levels of financial worries were slightly higher among people with probable COVID-19 infection than in those without, but these differences were nonsignificant at both COVID-19 assessments after accounting for all covariates and prepandemic financial situation (Fig. 1 and *SI Appendix*, Table S3). Lastly, no significant differences in the levels of social contact with family and friends were found between people with and without probable COVID-19 infection (Fig. 1 and *SI Appendix*, Table S3).

### Interaction Effects between Probable Infection and Sociodemographic Factors on Mental Health, Financial Hardship, and Social Interactions.

**Fig. 3.**
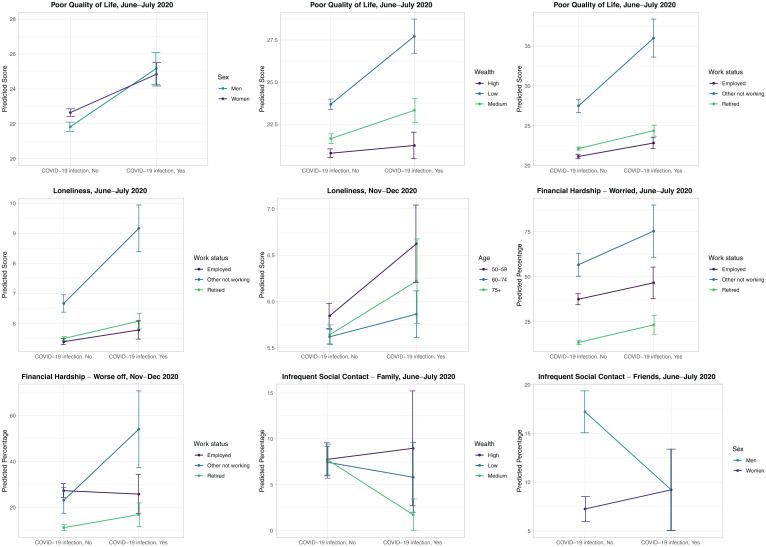
Interaction effects between probable COVID-19 infection and sociodemographic factors on wellbeing, financial hardship, and social connections. ELSA COVID-19 longitudinal sample (n = 5,146); pooled estimates and 95% CIs derived from logistic/linear regression models across 20 imputed datasets and weighted using survey weights; models adjusted for sex, age, pre-COVID-19 outcomes, whether living alone, employment status, wealth, whether vulnerable to COVID-19, November or December 2020 COVID-19 infection (November to December 2020 outcome only), and limiting long-standing illness and including interaction effects between COVID-19 infection and sociodemographic factors; only significant interaction effects (95% level) are reported.

The predicted outcome scores for the significant interaction effects (95% level) are shown in Fig. 3, and the full statistical results of the moderation analyses are reported in *SI Appendix*, Tables S4–S12. We found some differences in the associations between probable COVID-19 infection and the outcomes by work status, wealth, sex, and age (Fig. 3). In particular, the negative impact of probable COVID-19 infection on poor QoL (b interaction 3.71 [95% CI: 0.49; 6.92] *P* = 0.024), loneliness (b interaction 1.37 [95% CI: 0.27; 2.47] *P* = 0.015), and financial difficulties (OR interaction 3.57 [95% CI: 1.21; 10.47] *P* = 0.021) was larger among people who were unemployed, permanently sick/disabled, or looking after family/home (i.e., “other not working” category) than in those who were employed. In addition, the increase in financial worries associated with probable COVID-19 infection was larger among people who were retired than in those who were employed (OR interaction 2.77 [95% CI: 1.06; 7.27] *P* = 0.038). The association between probable infection and poorer QoL was also greater among people with low levels of wealth compared with those with higher wealth (b interaction 1.50 [95% CI: 0.16; 2.84] *P* = 0.028), while probable COVID-19 infection was related to a lower likelihood of infrequent contact with family for the group with medium wealth but not for those with high wealth (OR interaction 0.17 [95% CI: 0.04; 0.71] *P* = 0.015). With regard to sex differences, the negative impact of probable COVID-19 infection on poor QoL was lower among women than in men (b interaction −1.28 [95% CI: −2.48; −0.09] *P* = 0.035). In addition, probable COVID-19 infection was associated with a lower likelihood of infrequent contact with friends in men but not in women (OR interaction 2.26 [95% CI: 1.01; 5.04] *P* = 0.047). Lastly, the association between probable COVID-19 infection and loneliness was smaller among participants aged 60 to 74 y compared with the youngest group (52 to 59 y) (b interaction −0.77 [95% CI: −1.43; −0.10] *P* = 0.024) (Fig. 3). The relationships of probable infection with depression and anxiety were similar across different sociodemographic groups.

### Sensitivity Analyses.

#### (i) IPTW analyses.

A comparison of the associations between COVID-19 infection and mental health, financial hardship, and social connections found in the inverse probability of treatment weighting (IPTW) regression analyses versus standard logistic/linear regression analyses is presented in *SI Appendix*, Figure S2 and Table S13. The associations found in the IPTW regression analyses mirrored those of the linear/logistic regression analyses discussed in the previous section (*SI Appendix*, *SI Results*).

#### (ii) Correction for multiple testing.

To account for multiple testing, we adjusted the *P* values of all associations and interaction effects tested in the main analyses, using the false discovery rate (FDR) method. The pattern of relationships between probable infection and the outcomes was almost unchanged following the correction for multiple testing, except for anxiety levels, which were no longer significantly different between people with and without probable infection (*SI Appendix*, Table S3). In contrast, the interaction effects between probable infection and the sociodemographic factors did not remain after FDR correction (*SI Appendix*, Tables S4–S12), which could be a consequence of the relatively small magnitude of the interaction effects and the low number of participants classified as probable COVID-19 cases in our study.

#### (iii) Alternative definitions of COVID-19 infection.

Using stricter criteria to define probable infections resulted in 3.9% of participants classified as probable COVID-19 cases based on definition 2 (i.e., a positive COVID-19 test result, hospitalization due to COVID-19, or two of the three core symptoms of COVID-19) and 6.7% of participants based on definition 3 (i.e., a positive COVID-19 test result, hospitalization due to COVID-19, or one of two core symptoms of COVID-19, including a new continuous cough and loss of sense of smell or taste). In the regression analysis with definition 2, probable infection was associated only with higher ratings of poor QoL and loneliness and worse financial situation; when using definition 3, probable infection was related to an increased risk of depression, greater levels of poor QoL and loneliness, and worse financial situation (*SI Appendix*, Table S14). Of note, the limited number of participants classified as probable COVID-19 cases based on these definitions reduced the statistical power of this sensitivity analysis.

#### (iv) Real-time contact with family and friends.

We tested the impact of probable COVID-19 infection on social interactions considering only the amount of real-time contact that participants had with their family and friends (*SI Appendix*, Tables S15 and S16). As for the main analysis, no differences were observed in the levels of real-time social contact with family and friends between people with and without probable COVID-19 infection.

#### (v) Complete data analyses.

We restricted the analyses to participants with complete data on all variables. The associations of probable COVID-19 infection with the outcomes at each COVID-19 assessment (*SI Appendix*, Table S17) aligned closely with those of the main analysis with the imputed data.

#### (vi) Adjustment for and interactions with mental and physical health conditions.

No substantial differences were observed in the associations between probable COVID-19 infection and the outcomes when the analyses also accounted for the presence of a mental health condition at the first COVID-19 assessment or wave 9 and for the onset of new physical health conditions at the first COVID-19 assessment. In addition, we found no evidence for the moderating role of mental or physical health conditions in the associations between probable infection and the outcomes (*SI Appendix*, Tables S18–26).

#### (vii) Associations of prepandemic mental health with probable COVID-19 infection.

Lastly, we tested the association of prepandemic mental health (2018 to 2019) with probable COVID-19 infection to clarify whether the nature of their relationship might be bidirectional. Prepandemic depression (OR 1.94 [95% CI: 1.33; 2.85] *P* = 0.001) and higher poor QoL scores (OR 1.04 [95% CI: 1.02; 1.07] *P* < 0.001) were associated with a higher risk of probable COVID-19 infection in June to July 2020 (*SI Appendix*, Table S27).

## Discussion

### Summary of Main Findings.

Older adults with probable COVID-19 infection reported higher levels of depression and anxiety, poorer QoL, and greater loneliness scores compared with those without probable infection. These associations were independent of prepandemic differences in mental health and wellbeing, and they were evident both when assessed close to the presumed start of the infection (June to July 2020) and at the follow-up assessment (November to December 2020), thereby providing evidence for the longer-term psychological impact of contracting COVID-19. Older adults with probable infection also reported greater financial difficulties than those without infection in June and July 2020, but not at the follow-up assessment. In contrast, the levels of social contact with family and friends were similar in participants with and without probable infection, suggesting that the experience of COVID-19 symptoms was unrelated to changes in older adults' social relationships. People who were unemployed, permanently sick/disabled, or looking after the family/home were particularly vulnerable to the impact of probable infection on QoL, loneliness, and financial difficulties. QoL scores were also more negatively affected by probable infection in people with low levels of wealth than in wealthier participants. Lastly, compared with women, men reported lower QoL scores but higher social contact with friends if they probably had COVID-19. However, such differences in the associations of probable infection with wellbeing and financial outcomes across different sociodemographic groups were modest and did not remain following the correction for multiple testing.

### Interpretation of Findings.

#### (i) Mental health and wellbeing.

Earlier studies documenting the adverse psychological effects of COVID-19 infection have focused on small samples and lacked data on the participants’ mental health before the infection (5, 12–19). A large U.S. study using electronic health records found that COVID-19 patients with no previous psychiatric history were at increased risk of first-time diagnosis of psychiatric disorders compared with those affected by other health events (20). However, this assessed only severe mental ill-health resulting in medical consultation. Our study provides evidence of the longer-term association between probable COVID-19 infection and unfavorable mental health and wellbeing outcomes among older adults, indicating that the adverse impact of COVID-19 infection on mental health is more broadly present across the population. Of note, these associations were independent of prepandemic mental health and other confounding factors across different statistical approaches to control for confounding in observational research, thereby strengthening our confidence in the likely causal effect of COVID-19 infection on mental health. Our analysis also highlights the adverse impact that experiencing long COVID-19 might have on an individual’s mental health. Indeed, psychological distress is commonly reported 6 months after hospital discharge in severely infected people (5). However, we were unable to identify people with long COVID-19 in the present analysis since the ELSA COVID-19 substudy did not collect data on the duration of symptoms.

Another aspect of our analysis lies in the identification of population groups that might be particularly vulnerable to the psychological impact of contracting COVID-19. We found that older adults who are out of work, those with low levels of wealth, and men are at particular risk for low wellbeing if they probably contracted the infection. Similar socioeconomic and sex disparities in the impact of COVID-19 infection on wellbeing have also been found in other studies (23, 37). Taken together, these findings suggest that men and people with low socioeconomic status might be less resilient to the psychological effects of COVID-19 infection, and such inequalities could at least partly underlie their increased vulnerability to severe COVID-19 outcomes and mortality (38). In contrast, analyses of mental health and wellbeing across the whole population of older adults have found that women and people with high socioeconomic status have responded to the pandemic with more negative changes (39), suggesting that the experience of COVID-19 symptoms might modify these population-wide trends in mental health during the pandemic.

#### (ii) Financial hardship and social connections.

Despite the well-documented impact of the COVID-19 pandemic on older adults’ personal finances and social relationships (25, 28), longitudinal evidence on the impact that contracting the virus itself may have on these outcomes is scarce. Our analyses show that probable COVID-19 infection is related only to short-term increases in financial hardship among older adults living in the community. By the end of 2020, the heightened financial hardship of people with COVID-19 infection early in the pandemic had declined to the levels reported by the rest of the population. Nevertheless, people with probable infection who were unemployed, permanently sick/disabled, or looking after the family/home experienced greater financial hardship than those who were employed at both the first and second COVID-19 assessments. Hence, older adults who are out of work for reasons other than retirement might be at particular risk for longer-term financial difficulties following the infection.

Older adults’ levels of social contact with family and friends were generally unrelated to the experience of probable COVID-19 symptoms. Additionally, men who probably had COVID-19 reported greater levels of contact with friends compared with those who did not have the infection. Since social support is known to play a beneficial role in the recovery from physical and mental illnesses (40), this finding could indicate that older people with COVID-19 infection might either maintain their usual levels of social contact or make more contact with family and friends as a strategy to support their mental health during the recovery from the disease. However, it is important to note that the majority of our participants were living in the community. The situation is likely to be much worse for those living in nursing or care homes, as the regulations preventing visits from relatives might have had a profound effect on their social relationships (28).

### Strengths and Limitations.

This study has several strengths. The analyses used longitudinal data from a large, nationally representative sample of older adults. All outcomes were assessed before the COVID-19 pandemic began (i.e., 2019) and on two occasions during the pandemic (i.e., June to July and November to December 2020), which enabled us to explore both acute and longer-term responses to the infection and to account for differences present before the pandemic. The data were collected online and by telephone interview, and response rates were very high at both assessments. We used well-known measures of depression, anxiety, loneliness, and QoL, as well as multiple strategies to take account of confounding factors and assess the robustness of the results. However, the results presented here must be interpreted in light of their limitations. The classification of probable COVID-19 infection was based on self-reported symptoms of COVID-19 and was not confirmed by a laboratory test, so not all participants classified as suspected COVID-19 cases might have actually contracted the infection. Indeed, a range of infections and conditions other than COVID-19 could lead to fever and cough. Nevertheless, our sensitivity analyses show that the associations between probable infection and psychosocial outcomes are independent of the presence of a limiting longstanding illness or the onset of new health conditions at the first COVID-19 assessment. Symptoms of COVID-19 were ascertained only at the first COVID-19 assessment in June and July 2020; therefore, we could not determine the duration of symptoms and identify people with long COVID-19. Furthermore, the power of our analysis to identify population subgroups most vulnerable to the psychosocial consequences of contracting COVID-19 could be low due to the relatively small magnitude of the interaction effects and the low number of participants classified as probable COVID-19 cases. It is also worth noting that the levels of depression observed in our study were considerably higher than those of anxiety. This finding is in line with the results of a recent meta-analysis of longitudinal cohort studies showing that increases in depression during the COVID-19 pandemic have been almost twice as large and more persistent than increases in anxiety (41). Nevertheless, a potential limitation is that both depression and anxiety were assessed using shorter versions of the original Centre for Epidemiological Studies Depression (CESD) and GAD scales and may not fully capture all aspects of these disorders. Therefore, it would be important to understand whether similar results are also found when considering clinical diagnoses of depression and anxiety. Lastly, psychological factors are known to have bidirectional links with physical disease (42). As indicated by our sensitivity analyses, the relationship between probable COVID-19 infection and mental health is likely to be bidirectional, whereby people with worse prepandemic mental health could be at greater risk for COVID-19 infection.

### Conclusions.

Our study suggests that older people with probable COVID-19 infection are at particular risk for depression, anxiety, loneliness, and low wellbeing both in the acute phase of the infection and up to 6 months after the presumed start of the infection. Short-term increases in financial hardship were also observed, whereas levels of social contact with family and friends were generally unrelated to the experience of COVID-19 symptoms. Men, people with low levels of wealth, and those out of work were particularly vulnerable to the adverse psychosocial consequences of contracting the virus, although such differences were small. These findings underscore the need to monitor the mental health and wellbeing of older people affected by COVID-19 both in the acute and recovery phases of the disease, and they also highlight the importance of ensuring access to mental health support to those in need, particularly in the presence of prolonged COVID-19 symptoms. Additional financial support should be made available to support older adults’ physical and psychological recovery from COVID-19 infection.

## Materials and Methods

### Sample.

We analyzed data from the COVID-19 substudy of the ELSA, a longitudinal cohort study of men and women aged 50 y and older living in England (*SI Appendix*, *SI Methods*). In 2020, a COVID-19 substudy based on the regular ELSA sample was launched to investigate the socioeconomic and psychological impacts of the pandemic on the older population of England. The first assessment was completed in June and July 2020 and coincided with the later stages of the first infection peak, while the second assessment took place 5 months later (November to December 2020) during the period of increased infection and second national lockdown in the United Kingdom. The response rate was high in both COVID-19 assessments (75%), and the longitudinal response rate was 94.2% (43). Most participants were living in the community (only four participants were living in a care home at the first assessment). A sample of 5,146 core ELSA members of the COVID-19 substudy who participated in both COVID-19 assessments and in the most recent regular ELSA wave before the pandemic (i.e., wave 9 [2018 and 2019]) was employed. The statistical analyses were weighted using the longitudinal survey weights to account for nonresponse to the COVID-19 survey and match the latest population estimates for age, sex, housing tenure, relationship status, and region in England. A comparison of the characteristics of the analytical sample versus the regular ELSA sample is presented in *SI Appendix*, Table S1. All respondents provided informed consent. Ethical approval for the regular ELSA study was obtained from the National Research Ethics Service. The ELSA COVID-19 substudy has been approved by the University College London Research Ethics Committee. Further information can be found in the survey documentation (43) and on the study website (https://www.elsa-project.ac.uk/).

### Measures.

#### Outcomes.

We used assessments of depression and anxiety as measures of mental health and assessments of QoL and loneliness as measures of wellbeing. Depression was ascertained using the 8-item CESD (CESD-8) scale, with a cutoff point of four or more symptoms to identify likely cases of clinical depression. Anxiety was ascertained using the 7-item GAD scale (GAD-7), with a threshold score of 10 or greater to identify likely cases of generalized anxiety disorder. QoL was evaluated through the 12-item Control, Autonomy, Self-realization, and Pleasure (CASP) scale. The resulting item scores were summed to create a QoL index where higher scores indicate poorer wellbeing (range: 1 to 48). Loneliness was measured using the 3-item revised University of California loneliness scale and an additional item asking participants how often they feel lonely. The item scores were summed to derive a total score, with higher values indicating greater loneliness (range: 1 to 12) (see *SI Appendix*, *SI Methods* for further details). Financial hardship during the pandemic was measured using the following binary variables: 1) whether the participants are worried about their future financial situation (i.e., somewhat, very, or extremely worried versus not very or not at all worried) and 2) whether their financial situation is worse than before COVID-19 (i.e., a little or much worse versus about the same, a little better, or much better). To assess social interactions, participants were asked about the amount of real-time contact (i.e., by telephone or video calling) and written contact (i.e., emails, letters, texts) they had with their family outside the household and friends in the past month. We derived two binary variables (one for each source of support) indicating whether the respondents had infrequent contact with their family and friends. Infrequent contact was defined as having contact with family/friends less than once a week, as in an earlier ELSA study (44). We also calculated a continuous index of infrequent contact with family and friends by adding together the individual item scores, with higher scores indicating less frequent contact (range: 8 to 32). All outcomes were assessed repeatedly at both COVID-19 assessments.

#### Probable COVID-19 infection.

Serological testing for COVID-19 infection was not introduced in the United Kingdom in the early months of the pandemic, so there only were 10 participants who reported testing positive for COVID-19 at the first COVID-19 assessment. We therefore combined this information with data on self-reported COVID-19 symptoms in order to identify individuals with probable COVID-19 infection in June and July 2020. The following criteria were applied to define probable COVID-19 infection. Under definition 1, participants were found to be COVID-19 positive on serological testing, were hospitalized due to COVID-19, or reported one of the three core symptoms as defined by the U.K. National Health Service (NHS) (i.e., high temperature, a new continuous cough, and loss of sense of smell or taste) (45). A similar approach to defining probable COVID-19 infection has been previously used in other population-based studies (23). As a sensitivity analysis, we used two alternative definitions of probable COVID-19 symptoms. Under definition 2, participants were found to be COVID-19 positive on testing, were hospitalized due to COVID-19, or reported at least two of the three core symptoms identified by the NHS; and under definition 3, participants were found to be COVID-19 positive on testing, were hospitalized due to COVID-19, or reported at least one of two core symptoms of COVID-19 infection, including a new continuous cough and loss of sense of smell or taste but excluding fever, as the latter could be a symptom of many other diseases/infections other than COVID-19.

#### Covariates.

Prepandemic mental health, social contact, and financial difficulties were included as covariates to account for differences between people with and without probable COVID-19 infection before the onset of the pandemic. Covariates obtained from the first COVID-19 assessment included sex, age, whether or not they were living alone, work status, and whether or not they were vulnerable to COVID-19 infection. The analyses of the outcomes at the second COVID-19 assessment also accounted for whether participants reported testing positive or being hospitalized for COVID-19 in November and December 2020 (information on COVID-19 symptoms was not collected in the second COVID-19 assessment). Additional covariates taken from the wave 9 survey were wealth and limiting long-standing illness. The variables sex, age, living alone, work status, and wealth were also considered as possible effect modifiers in the analysis in order to explore whether the psychosocial impact of COVID-19 infection might vary across distinct sociodemographic groups (see *SI Appendix*, *SI Methods* for further information).

### Statistical Analyses.

The associations of probable infection (first COVID-19 assessment) with mental health, financial hardship, and social interactions at the first (immediate impact) and second (longer-term impact) COVID-19 assessments were tested using linear or logistic regression analysis. These analyses were adjusted for all covariates, including the prepandemic scores of the outcomes to account for preexisting differences between participants with and without probable COVID-19 infection, and weighted using the longitudinal survey weights. The results for categorical outcomes are reported as adjusted ORs, and those for continuous outcomes as adjusted beta (b) coefficients, with 95% CIs. Estimated proportions and means of the outcomes for people with and without probable COVID-19 infection adjusted for all covariates are also shown. We assessed whether the associations of probable COVID-19 infection with the outcomes varied across different sociodemographic groups using interaction effects with five sociodemographic factors (i.e., sex, age, living alone, work status, and wealth). Missing data on all variables were estimated using multiple imputation by chained equations (MICE). In sensitivity analyses, we tested the associations between probable COVID-19 infection and the outcomes using IPTW, also known as propensity score weighting. Further details regarding MICE, IPTW and other sensitivity analyses, and the statistical software are described in *SI Appendix*, *SI Methods*.

## Supplementary Material

Supplementary File

## Data Availability

Data from ELSA can be accessed through the U.K. data service (https://ukdataservice.ac.uk/). The code of the statistical analyses can be accessed on GitHub (https://github.com/Ellie25moon/ELSA-COVID-19-Infection) (46). The data used in this work can be obtained free upon registration at the U.K. data service https://beta.ukdataservice.ac.uk/datacatalogue/series/series?id=200011 (47). Further information regarding the sample design and data collection methods can be found on the study website (https://www.elsa-project.ac.uk/).
