# Supplementary material for: Mental health, financial, and social outcomes among older adults with probable COVID-19 infection: A longitudinal cohort study
Source: Proc Natl Acad Sci U S A. 2022 Jun 28;119(27):e2200816119. doi: 10.1073/pnas.2200816119 (PMC9271189; doi:10.1073/pnas.2200816119)
Supplement: Supplementary File [file pnas.2200816119.sapp.pdf]

PNAS
www.pnas.org

Supporting Information for

Mental health, financial, and social outcomes among older adults with probable COVID-19 infection: A longitudinal cohort study.

Eleonora Iob, PhD

Andrew Steptoe, DSc

Paola Zaninotto, PhD

Corresponding author: Eleonora Iob, email: eleonora.iob.17@ucl.ac.uk.

This PDF file includes:

sTable1 to sTable18
sFigure1 to sFigure2

sMethods

Sample

The English Longitudinal Study of Ageing (ELSA) is a longitudinal cohort study of men and women aged 50 years and older living in England. The study commenced in 2002/03 (wave 1) with a nationally representative sample of 12,099 participants and their partners, drawn from the Health Survey for England (HSE). To record changes in their health, social, and economic circumstances, since the commencement of the study these participants have been repeatedly interviewed at biennial interviews (i.e. waves) using face-to-face computer-assisted personal interviews and self-completion questionnaires. The most recent full wave of data collection was carried out in 2018/19 (wave 9). The study sample is periodically refreshed with new participants to maintain its original size and representativeness.

Measures

Mental health outcomes

Depression was ascertained using the 8-item Centre for Epidemiological Studies Depression (CESD-8) scale(1). We used a cut-off point of four or more symptoms to identify likely cases of clinical depression(2). Anxiety was ascertained using the 7-item Generalised Anxiety Disorder scale (GAD-7)(3), with a threshold score of 10 or greater to identify likely cases of generalised anxiety disorder (GAD). Quality of life (QoL) was evaluated through the 12-item Control, Autonomy, Self-realisation, and Pleasure (CASP) scale(4). The resulting item scores were summed to create a QoL index where higher scores indicate poorer wellbeing (range: 1-48). Loneliness was measured using the 3-item revised University of California (UCLA) Loneliness scale(5) and an additional item asking participants how often they feel lonely. The item scores were summed to derive a total score, with higher values indicating greater loneliness (range: 1-12).

Covariates

Pre-pandemic mental health, social contact, and financial difficulties were included as covariates to account for differences between people with and without probable COVID-19 infection before the onset of the pandemic. Pre-pandemic mental health and social interactions were measured at the most recent pre-pandemic assessment available for each participant (i.e. wave 9). Assessments of depression, QoL, and loneliness in earlier waves of ELSA were conducted using the same scales as those included in the COVID-19 substudy. The GAD-7 was not administered in previous waves; to adjust the analyses for anxiety levels pre-pandemic we used the Office for National Statistics (ONS) anxiety scale administered in wave 9. The anxiety scale is one of the four wellbeing measures that have been developed by the Office for National Statistics (ONS) to assess various domains of personal wellbeing, including evaluative, eudemonic, and affective experience(6). Participants were asked to respond to the following question: "On a scale where 0 is 'not at all anxious' and 10 is 'completely anxious', overall, how anxious did you feel yesterday?". The resulting scores range between 0 and 10, with higher scores indicating greater anxiety levels. Further, as the social contact measures of the COVID-19 substudy differed from those included in earlier waves, the analyses of social interactions were adjusted for an index of social isolation combining information on whether participants had infrequent contact with family and friends and were not involved in local organisations at wave 9. Social isolation before the onset of the pandemic was measured using

four indicators that were obtained from the wave 9 survey. These included having less than monthly contact (face-to-face, telephone, or writing/email) with (i) children, (ii) other family, and (iii) friends, and (iv) no membership of nine types of organisation (i.e. a political party, trade union, or environmental group; a tenants groups, resident groups, neighbourhood watch; a church or other religious group; a charitable association; an education, arts or music groups or evening class; a social club; a sports clubs, gym, or exercise class; any other organisation, club or society). This measure of social isolation has been previously used in earlier ELSA studies(7, 8). Pre-pandemic financial hardship was measured based on the participants' ratings of their financial situation in the three months prior to the COVID-19 outbreak (i.e. "living comfortably", "doing all right", "just about getting by", "finding it quite difficult", or "finding it very difficult"). Covariates obtained from the first COVID-19 assessment included sex, age (52-59, 60-74, and 75+ years), whether living alone, work status (employed, retired, other not working), and whether vulnerable to COVID-19 infection (i.e. participants advised by the NHS or their general practitioner that they are at risk of severe illness if they catch COVID-19 and encouraged to stay at home and avoid face-to-face contact). The analyses of the outcomes at the second COVID-19 assessment also accounted for whether participants reported testing positive or being hospitalised for COVID-19 in Nov-Dec 2020 (information on COVID-19 symptoms was not collected in the second COVID-19 assessment). Additional covariates taken from the wave 9 survey were wealth and limiting long-standing illness. Wealth was used as an indicator of the participant's socioeconomic resources, including financial, housing, and physical wealth (such as land, business wealth, and jewellery) but excluding pension wealth, and it was divided into tertiles for the purpose of the analysis (i.e. low, medium, and high wealth). Participants were classified as having a limiting long-standing illness if they reported having a long-standing illness, disability, or infirmity that limited their activities in any way. The variables sex, age, living alone, work status, and wealth were also considered as possible effect modifiers in the analysis in order to explore whether the psychosocial impact of COVID-19 infection might vary across distinct sociodemographic groups.

Statistical analyses

Multiple imputation of missing data

The percentage of missing data in the variables ranged between 0 and 6%. In addition, due to a coding error, for around 75% of the sample the last item of the CESD-8 questionnaire was not administered at the first COVID-19 wave. This type of missing data is classified as missing completely at random (MCAR), and can be dealt efficiently with multiple imputation(9). We used multiple imputation by chained equations with all variables of the analysis included as predictors of the imputation models as well as auxiliary variables. We created twenty imputed datasets. The distribution of the variables in the imputed and observed data was similar, suggesting that the MI procedure produced accurate model estimates. In regression analyses, we pooled the regression estimates across the imputed datasets using Rubin's rules(10). For the IPTW analyses, propensity score estimation and weighting were performed within each imputed dataset, and the regression estimates across the weighted datasets were then pooled together – i.e. 'within' approach(11). We favoured this approach over the 'across' approach (i.e. propensity scores are averaged across the imputed datasets, and weighting within each dataset is performed using this averaged measure) because the latter has been shown to have inferior statistical performance compared with the 'within' approach across different scenarios(12, 13).

Sensitivity analyses

The following analyses were conducted:

- We applied inverse probability of treatment weighting (IPTW) to account for measured confounding factors in the associations between probable COVID-19 infection and the outcomes.
- To account for multiple testing, we corrected the p-values of all associations tested in the main analyses using the false discovery rate (FDR) method.
- We tested the associations between probable COVID-19 infection and the outcomes using two alternative definitions of probable COVID-19 symptoms (see Manuscript, 'Measures').
- We evaluated the associations of probable infection with social interactions considering only the amount of real-time contact that participants had with their family and friends.
- The regression models of the main imputed data analysis were tested again in a subsample of participants with complete data on all variables.
- We further adjusted the associations of probable COVID-19 infection with the outcomes for the presence of a mental health condition (either at the first COVID-19 assessment or at wave 9) and for the development of new physical health conditions at the first COVID-19 assessment, including cardiovascular conditions (e.g. hypertension, stroke), diabetes, respiratory illnesses (e.g. chronic lung disease, asthma), arthritis, cancer, and diseases of the visual, digestive, and nervous systems. In addition, we examined the interactions between these two variables and probable COVID-19 infection on all outcomes.
- Lastly, we tested the associations of pre-pandemic levels of depression, anxiety, poor QoL, and loneliness (2018-19) with probable COVID-19 infection (June-July 2020) in order to clarify whether the nature of their relationship might be bidirectional.

Inverse probability of treatment weighting (IPTW)

This technique provides unbiased estimates of the average treatment effect of an exposure on the outcome independent of measured baseline confounding factors, and it enabled us to understand how the results compare across different strategies to account for confounding in observational studies(14). The propensity scores were estimated with logistic regression models, including all covariates, the pre-pandemic outcome scores, and the longitudinal survey weights as predictors of the probability of COVID-19 infection. The balance of the covariates across the exposure groups in the unweighted and weighted datasets was assessed by computing the standardized mean differences (SMDs) for each covariate. Although no threshold is universally accepted, covariates with SMDs lower than 0.1 are typically considered well balanced(15).

Software

Data management and linear/logistic regression analyses were conducted in Stata 16. Graphical(16), MICE(17), and IPTW(11) analyses were performed in R version 4.0.2.

sResults

Descriptive statistics

The distribution of the covariates and outcomes in participants with and without probable COVID-19 infection at the first COVID-19 assessment is shown in Table 1. Participants with probable COVID-19 infection were younger on average, were more likely to be employed than to be retired, and had lower wealth compared with those without probable infection. People with probable COVID-19 infection were also more likely to experience elevated symptoms of depression and anxiety; they reported poorer quality of life and loneliness; and they were more worried about their future financial situation and more likely to experience greater financial difficulties than before COVID-19. The levels of infrequent contact with family and friends were slightly lower in people with probable COVID-19 infection than in those without, but these differences were not statistically significant (Table 1). Similar differences in mental health and financial hardship between participants with and without COVID-19 infection were also observed at the second COVID-19 assessment (SI – sTable2).

Sensitivity analyses

(i) IPTW analyses

A comparison of the associations between COVID-19 infection and mental health, financial hardship, and social connections found in the IPTW regression analyses versus standard logistic/linear regression analyses is presented in sFigure2 (SI). The full statistical results of the IPTW regression models are reported in sTable13 (SI). The balance of the covariates across the exposure groups in the propensity score weighted and unweighted datasets is shown in sFigure1 (SI). SMDs for all covariates were lower than 0.1 in the weighted sample. The associations found in the IPTW regression analyses mirrored those of the main linear/logistic regression analyses. Compared with those without probable COVID-19 infection, people with probable infection were at increased risk of depression ($OR_{assess1}$ 1.36 [95%CI: 1.08;1.71] p=0.010; $OR_{assess2}$ 1.47 [95%CI: 1.19;1.81] p<0.001), anxiety ($OR_{assess1}$ 1.59 [95%CI: 1.17;2.17] p=0.003; $OR_{assess2}$ 1.69 [95%CI: 1.27;2.24] p<0.001), poor QoL ($b_{assess1}$ 0.93 [95%CI: 0.24;1.62] p=0.008; $b_{assess2}$ 1.33 [95%CI: 0.64;2.02] p<0.001), and loneliness ($b_{assess1}$ 0.38 [95%CI: 0.15;0.61] p=0.001; $b_{assess2}$ 0.24 [95%CI: 0.01;0.48] p=0.039) at both COVID-19 assessments, and they also were more likely to experience greater financial difficulties in June-July 2020 than before the start of the pandemic ($OR_{assess1}$ 1.40 [95%CI: 1.11;1.77] p=0.005). In contrast, no significant differences in the levels of financial worries and social contact with family and friends were observed between people with and without probable infection (Figure 2; SI – sTable13).

References

1. L. S. Radloff, The CES-D Scale. *Appl. Psychol. Meas.* **1**, 385–401 (1977).
2. D. Steffick, "Documentation of Affective Functioning Measures in the Health and Retirement Study" (2000) (July 8, 2020).
3. R. L. Spitzer, K. Kroenke, J. B. W. Williams, B. Löwe, A brief measure for assessing generalized anxiety disorder: The GAD-7. *Arch. Intern. Med.* **166**, 1092–1097 (2006).
4. R. D. Wiggins, G. Netuveli, M. Hyde, P. Higgs, D. Blane, The Evaluation of a Self-enumerated Scale of Quality of Life (CASP-19) in the Context of Research on Ageing: A Combination of Exploratory and Confirmatory Approaches. *Soc. Indic. Res.* **89**, 61–77 (2007).
5. M. E. Hughes, L. J. Waite, L. C. Hawkley, J. T. Cacioppo, A Short Scale for Measuring Loneliness in Large Surveys: Results From Two Population-Based Studies. *Res. Aging* **26**, 655–672 (2004).
6. Office for National Statistics, "Surveys using our four personal well-being questions" (2018).
7. A. Steptoe, A. Shankar, P. Demakakos, J. Wardle, Social isolation, loneliness, and all-cause mortality in older men and women. *Proc. Natl. Acad. Sci.* **110**, 5797–5801 (2013).
8. A. Steptoe, G. Di Gessa, Mental health and social interactions of older people with physical disabilities in England during the COVID-19 pandemic: a longitudinal cohort study. *Lancet Public Heal.* (2021) https:/doi.org/10.1016/S2468-2667(21)00069-4 (April 27, 2021).
9. I. Eekhout, *et al.*, Missing data in a multi-item instrument were best handled by multiple imputation at the item score level. *J. Clin. Epidemiol.* **67**, 335–342 (2014).
10. D. B. Rubin, *Multiple Imputation for Nonresponse in Surveys* (John Wiley and Sons, 2004).
11. F. Pishgar, N. Greifer, C. Leyrat, E. Stuart, MatchThem:: Matching and Weighting after Multiple Imputation. *arXiv* (2020).
12. B. Penning de Vries, R. Groenwold, Comments on propensity score matching following multiple imputation. *Stat. Methods Med. Res.* **25**, 3066–3068 (2016).
13. C. Leyrat, *et al.*, Propensity score analysis with partially observed covariates: How should multiple imputation be used? *Stat. Methods Med. Res.* **28**, 3–19 (2019).
14. P. C. Austin, An introduction to propensity score methods for reducing the effects of confounding in observational studies. *Multivariate Behav. Res.* **46**, 399–424 (2011).
15. J. S. Haukoos, R. J. Lewis, The Propensity Score. *JAMA* **314**, 1637 (2015).
16. H. Wickham, *ggplot2: Elegant Graphics for Data Analysis* (Springer-Verlag, 2016).
17. S. van Buuren, K. Groothuis-Oudshoorn, mice: Multivariate Imputation by Chained Equations inR. *J. Stat. Softw.* **45** (2011).

Supplementary tables and figures

sTable1. Comparison of the characteristics of ELSA participants included in the analytical sample versus those not included at wave 9.			
	**Non-included (N=3590)**	**Included (N=5146)**	**p-value**
**Age**			0.004
Mean (SD)	67.566 (11.275)	68.207 (9.045)	
Range	50.000 - 90.000	50.000 - 90.000	
**Sex**			0.016
Male	1639 (45.7%)	2215 (43.0%)	
Female	1951 (54.3%)	2931 (57.0%)	
**Ethnicity**			< 0.001
White	3343 (93.3%)	4955 (96.3%)	
Non-White	241 (6.7%)	189 (3.7%)	
**Work status**			< 0.001
Retired	1845 (51.4%)	3305 (64.2%)	
Employed	1323 (36.9%)	1437 (27.9%)	
Other not working	422 (11.8%)	404 (7.9%)	
**Wealth**			0.014
Mean (SD)	189634.083 (525585.754)	217683.739 (512556.189)	
Range	-55000.000 - 12416100.000	-100900.000 - 12416100.000	
**Self-rated health**			< 0.001
Excellent	404 (12.9%)	629 (12.4%)	
Very good	718 (23.0%)	1653 (32.6%)	
Good	1006 (32.2%)	1699 (33.6%)	
Fair	664 (21.3%)	794 (15.7%)	
Poor	332 (10.6%)	289 (5.7%)	
Note. *P-values obtained from t-tests (continuous variables) or chi-square tests (categorical variables); SD = standard deviation.*			

sTable2. Mental health, financial hardship, and social connections in people with and without probable COVID-19 infection at the second COVID-19 assessment (Nov-Dec 2020).			
	**Probable infection: No (N=4,645)**	**Probable infection: Yes (N=501)**	**P-value**
**Elevated depressive symptoms (CESD-8 ≥ 4)**			
No	73.00%	58.20%	<0.001
Yes	27.00%	41.80%	
**Anxiety (Gad-7 ≥ 10)**			
No	89.80%	82.80%	0.001
Yes	10.20%	17.20%	
**Poor Quality of Life (CASP-12)**			
Mean (SD)	22.785 (6.572)	25.631 (7.018)	<0.001
Range	9.000 - 48.000	12.000 - 45.000	
**Loneliness**			
Mean (SD)	5.694 (2.126)	6.253 (2.510)	0.001
Range	3.000 - 12.000	4.000 - 12.000	
**Worried about future financial situation**			
No	73.90%	60.10%	<0.001
Yes	26.10%	39.90%	
**Current financial situation worse than pre-covid**			
No	81.60%	74.20%	0.009
Yes	18.40%	25.80%	
**Infrequent contact with family**			
No	89.70%	89.70%	0.977
Yes	10.30%	10.30%	
**Infrequent contact with friends**			
No	84.90%	82.70%	0.376
Yes	15.10%	17.30%	
**Infrequent contact with family and friends (total score)**			
Mean (SD)	24.589 (5.041)	24.429 (5.116)	0.641
Range	8.000 - 32.000	8.000 - 32.000	

Note. *Results based on 20 imputed datasets; percentages and means are estimated using sampling weights; pooled p-value estimates from logistic regression models across the imputed datasets, weighted using survey weights; SD = standard deviation.*

**sTable3. Associations between probable COVID-19 infection and mental health, financial hardship, and social connections (linear/logistic regression analyses).**						
**Outcome**	**Exposure**	**OR/b**	**p-value**	**CI (lower)**	**CI (upper)**	**FDR corrected p-value**
Mental Health	COVID-19 infection (vs no infection)					
Depression, June-July 2020 (OR)		1.617	**0.005**	1.158	2.258	**0.025**
Depression, Nov-Dec 2020 (OR)		1.564	**0.003**	1.169	2.092	**0.015**
Anxiety, June-July 2020 (OR)		1.586	**0.049**	1.003	2.508	0.184
Anxiety, Nov-Dec 2020 (OR)		1.553	**0.041**	1.018	2.370	0.160
Poor QoL, June-July 2020 (b)		1.340	**0.000**	0.661	2.018	**0.001**
Poor QoL, Nov-Dec 2020 (b)		1.382	**0.000**	0.737	2.027	**0.000**
Loneliness, June-July 2020 (b)		0.491	**0.000**	0.247	0.735	**0.001**
Loneliness, Nov-Dec 2020 (b)		0.310	**0.024**	0.042	0.577	0.100
Financial Hardship						
Worried, June-July 2020 (OR)		1.196	0.318	0.842	1.699	0.619
Worried, Nov-Dec 2020 (OR)		1.112	0.571	0.770	1.606	0.829
Worse off, June-July 2020 (OR)		1.502	**0.011**	1.099	2.053	**0.053**
Worse off, Nov-Dec 2020 (OR)		1.156	0.407	0.821	1.629	0.698
Social Connections						
Infrequent contact with family, June-July 2020 (OR)		0.698	0.184	0.410	1.187	0.432
Infrequent contact with family, Nov-Dec 2020 (OR)		0.998	0.991	0.649	1.533	0.994
Infrequent contact with friends, June-July 2020 (OR)		0.793	0.259	0.530	1.187	0.544
Infrequent contact with friends, Nov-Dec 2020 (OR)		1.252	0.248	0.855	1.835	0.538
Infrequent contact with family/friends (total score), June-July 2020 (b)		-0.178	0.483	-0.676	0.320	0.772
Infrequent contact with family/friends (total score), Nov-Dec 2020 (b)		0.039	0.871	-0.433	0.511	0.978
Note. *Sample: ELSA COVID-19 Substudy (N=5,146); pooled estimates from linear/logistic regression models across 20 imputed datasets, weighted using survey weights; Models adjusted for sex, age groups, pre-COVID-19 outcome, Nov-Dec 2020 COVID-19 infection (Nov-Dec 2020 outcome only), whether living alone, employment status, wealth, whether vulnerable to COVID-19, limiting long-standing illness; June-July 2020 = COVID-19 assessment 1; Nov-Dec 2020 = COVID-19 assessment 2; OR = odds ratio; CI = 95% confidence interval; QoL = quality of life; FDR = false discovery rate.*						

sTable4. Interaction effects between probable COVID-19 infection and sociodemographic factors on depression.						
**Outcome**	**Exposure**	**OR**	**p-value**	**CI (lower)**	**CI (upper)**	**FDR corrected p-value**
Depression, June-July 2020	COVID-19 infection	0.983	0.971	0.387	2.498	0.981
	Sex: Female (vs Male)	1.645	**0.000**	1.316	2.056	**0.000**
	COVID-19 infection*Female	1.162	0.668	0.584	2.314	0.884
	Age group: 60-74 (vs 52-59)	0.753	0.082	0.547	1.037	0.264
	Age group: 75+	0.525	**0.001**	0.359	0.769	**0.006**
	COVID-19 infection*60-74	0.827	0.741	0.268	2.550	0.937
	COVID-19 infection*75+	0.922	0.901	0.258	3.297	0.978
	Work: Retired (vs employed)	1.221	0.173	0.916	1.626	0.418
	Work: Other not working	2.677	**0.000**	1.735	4.131	**0.000**
	COVID-19 infection*Retired	1.687	0.353	0.559	5.085	0.653
	COVID-19 infection*Other not working	1.527	0.546	0.386	6.049	0.810
	Wealth: Medium (vs High)	1.157	0.285	0.885	1.513	0.586
	Wealth: Low	1.150	0.318	0.874	1.513	0.619
	COVID-19 infection*Medium wealth	1.063	0.877	0.492	2.293	0.978
	COVID-19 infection*Low wealth	1.339	0.458	0.619	2.899	0.751
	Living alone	1.518	**0.000**	1.212	1.901	**0.002**
	COVID-19 infection*Living alone	1.273	0.529	0.600	2.701	0.807
Depression, Nov-Dec 2020	COVID-19 infection	1.436	0.382	0.638	3.230	0.678
	Sex: Female (vs Male)	1.466	**0.000**	1.210	1.776	**0.001**
	COVID-19 infection*Female	1.120	0.704	0.623	2.014	0.906
	Age group: 60-74 (vs 52-59)	0.800	0.126	0.601	1.065	0.340
	Age group: 75+	0.533	**0.000**	0.383	0.742	**0.002**
	COVID-19 infection*60-74	0.521	0.170	0.206	1.322	0.416
	COVID-19 infection*75+	0.703	0.535	0.231	2.142	0.807
	Work: Retired (vs employed)	1.095	0.496	0.843	1.423	0.774
	Work: Other not working	2.285	**0.000**	1.535	3.401	**0.001**
	COVID-19 infection*Retired	1.514	0.379	0.600	3.818	0.678
	COVID-19 infection*Other not working	1.384	0.619	0.384	4.989	0.859
	Wealth: Medium (vs High)	1.105	0.401	0.875	1.396	0.696
	Wealth: Low	1.318	**0.025**	1.035	1.678	0.104
	COVID-19 infection*Medium wealth	1.294	0.481	0.631	2.653	0.772
	COVID-19 infection*Low wealth	1.174	0.674	0.554	2.488	0.884
	Living alone	1.542	**0.000**	1.250	1.904	**0.001**
	COVID-19 infection*Living alone	0.972	0.937	0.482	1.961	0.980

Note. *Sample: ELSA COVID-19 Substudy (N=5,146); pooled estimates from linear/logistic regression models across 20 imputed datasets, weighted using survey weights; models adjusted for sex, age, pre-COVID-19 outcome, Nov-Dec 2020 COVID-19 infection (Nov-Dec 2020 outcome only), whether living alone, employment status, wealth, whether vulnerable to COVID-19, and limiting long-standing illness and including interaction effects between COVID-19 infection and sociodemographic factors; June-July 2020 = COVID-19 assessment 1; Nov-Dec 2020 = COVID-19 assessment 2; OR = odds ratio; CI = 95% confidence interval; FDR = false discovery rate.*

**sTable5. Interaction effects between probable COVID-19 infection and sociodemographic factors on anxiety.**						
**Outcome**	**Exposure**	**OR**	**p-value**	**CI (lower)**	**CI (upper)**	**FDR corrected p-value**
Anxiety, June-July 2020	COVID-19 infection	0.473	0.301	0.115	1.952	0.601
	Sex: Female (vs Male)	1.348	0.082	0.962	1.888	0.264
	COVID-19 infection*Female	1.196	0.721	0.448	3.196	0.924
	Age group: 60-74 (vs 52-59)	0.740	0.209	0.463	1.184	0.464
	Age group: 75+	0.372	**0.000**	0.214	0.648	**0.003**
	COVID-19 infection*60-74	2.401	0.254	0.533	10.820	0.543
	COVID-19 infection*75+	1.802	0.494	0.333	9.737	0.774
	Work: Retired (vs employed)	1.569	**0.050**	1.000	2.463	0.186
	Work: Other not working	3.332	**0.000**	1.911	5.810	**0.000**
	COVID-19 infection*Retired	0.878	0.876	0.172	4.494	0.978
	COVID-19 infection*Other not working	0.938	0.933	0.212	4.147	0.980
	Wealth: Medium (vs High)	1.356	0.160	0.886	2.076	0.408
	Wealth: Low	1.919	**0.002**	1.269	2.902	**0.012**
	COVID-19 infection*Medium wealth	1.580	0.478	0.447	5.588	0.772
	COVID-19 infection*Low wealth	1.876	0.296	0.576	6.112	0.598
	Living alone	1.198	0.287	0.859	1.672	0.587
	COVID-19 infection*Living alone	1.646	0.384	0.536	5.060	0.678
Anxiety, Nov-Dec 2020	COVID-19 infection	0.321	0.123	0.076	1.359	0.339
	Sex: Female (vs Male)	1.635	**0.003**	1.184	2.258	**0.016**
	COVID-19 infection*Female	2.046	0.103	0.866	4.833	0.313
	Age group: 60-74 (vs 52-59)	0.533	**0.004**	0.348	0.814	**0.021**
	Age group: 75+	0.237	**0.000**	0.141	0.400	**0.000**
	COVID-19 infection*60-74	2.497	0.176	0.663	9.402	0.421
	COVID-19 infection*75+	3.123	0.156	0.647	15.077	0.401
	Work: Retired (vs employed)	2.293	**0.000**	1.535	3.424	**0.001**
	Work: Other not working	4.019	**0.000**	2.393	6.751	**0.000**
	COVID-19 infection*Retired	1.226	0.750	0.350	4.299	0.938
	COVID-19 infection*Other not working	1.555	0.541	0.377	6.412	0.809
	Wealth: Medium (vs High)	1.293	0.189	0.881	1.898	0.440
	Wealth: Low	1.425	0.062	0.982	2.069	0.218
	COVID-19 infection*Medium wealth	0.921	0.889	0.290	2.923	0.978
	COVID-19 infection*Low wealth	1.471	0.519	0.456	4.750	0.794
	Living alone	0.934	0.698	0.661	1.319	0.902
	COVID-19 infection*Living alone	1.642	0.303	0.639	4.214	0.601

Note. *Sample: ELSA COVID-19 Substudy (N=5,146); pooled estimates from linear/logistic regression models across 20 imputed datasets, weighted using survey weights; models adjusted for sex, age, pre-COVID-19 outcome, Nov-Dec 2020 COVID-19 infection (Nov-Dec 2020 outcome only), whether living alone, employment status, wealth, whether vulnerable to COVID-19, and limiting long-standing illness and including interaction effects between COVID-19 infection and sociodemographic factors; June-July 2020 = COVID-19 assessment 1; Nov-Dec 2020 = COVID-19 assessment 2; OR = odds ratio; CI = 95% confidence interval; FDR = false discovery rate.*

sTable6. Interaction effects between probable COVID-19 infection and sociodemographic factors on poor QoL.						
**Outcome**	**Exposure**	**b**	**p-value**	**CI (lower)**	**CI (upper)**	**FDR corrected p-value**
Poor QoL, June-July 2020	COVID-19 infection	0.887	0.201	-0.472	2.246	0.451
	Sex: Female (vs Male)	0.718	**0.000**	0.363	1.072	**0.001**
	COVID-19 infection*Female	-1.284	**0.035**	-2.477	-0.091	0.139
	Age group: 60-74 (vs 52-59)	-0.513	0.054	-1.035	0.010	0.195
	Age group: 75+	-0.312	0.363	-0.984	0.360	0.663
	COVID-19 infection*60-74	-1.280	0.145	-3.002	0.443	0.375
	COVID-19 infection*75+	-0.926	0.422	-3.186	1.335	0.707
	Work: Retired (vs employed)	0.386	0.112	-0.090	0.863	0.323
	Work: Other not working	1.985	**0.000**	1.032	2.938	**0.001**
	COVID-19 infection*Retired	1.091	0.211	-0.619	2.801	0.464
	COVID-19 infection*Other not working	3.705	**0.024**	0.495	6.916	0.100
	Wealth: Medium (vs High)	-0.123	0.536	-0.514	0.268	0.807
	Wealth: Low	0.051	0.815	-0.373	0.475	0.967
	COVID-19 infection*Medium wealth	0.771	0.193	-0.389	1.932	0.444
	COVID-19 infection*Low wealth	1.499	**0.028**	0.159	2.840	0.116
	Living alone	0.165	0.410	-0.227	0.557	0.698
	COVID-19 infection*Living alone	0.078	0.919	-1.424	1.580	0.979
Poor QoL, Nov-Dec 2020	COVID-19 infection	1.022	0.196	-0.527	2.572	0.449
	Sex: Female (vs Male)	0.740	**0.000**	0.379	1.102	**0.001**
	COVID-19 infection*Female	0.178	0.781	-1.076	1.432	0.958
	Age group: 60-74 (vs 52-59)	-0.857	**0.002**	-1.393	-0.321	**0.011**
	Age group: 75+	-0.587	0.121	-1.329	0.155	0.339
	COVID-19 infection*60-74	-0.958	0.324	-2.864	0.948	0.619
	COVID-19 infection*75+	-0.113	0.923	-2.403	2.177	0.980
	Work: Retired (vs employed)	0.691	**0.004**	0.215	1.168	**0.024**
	Work: Other not working	1.676	**0.001**	0.683	2.669	**0.006**
	COVID-19 infection*Retired	-0.170	0.850	-1.925	1.585	0.978
	COVID-19 infection*Other not working	0.614	0.667	-2.185	3.412	0.884
	Wealth: Medium (vs High)	0.089	0.660	-0.308	0.486	0.883
	Wealth: Low	0.127	0.564	-0.305	0.560	0.824
	COVID-19 infection*Medium wealth	1.085	0.131	-0.325	2.495	0.348
	COVID-19 infection*Low wealth	0.920	0.253	-0.659	2.499	0.543
	Living alone	0.163	0.441	-0.251	0.577	0.726
	COVID-19 infection*Living alone	-0.256	0.732	-1.716	1.205	0.930

Note. *Sample: ELSA COVID-19 Substudy (N=5,146); pooled estimates from linear/logistic regression models across 20 imputed datasets, weighted using survey weights; models adjusted for sex, age, pre-COVID-19 outcome, Nov-Dec 2020 COVID-19 infection (Nov-Dec 2020 outcome only), whether living alone, employment status, wealth, whether vulnerable to COVID-19, and limiting long-standing illness and including interaction effects between COVID-19 infection and sociodemographic factors; June-July 2020 = COVID-19 assessment 1; Nov-Dec 2020 = COVID-19 assessment 2; CI = 95% confidence interval; QoL = quality of life; FDR = false discovery rate.*

sTable7. Interaction effects between probable COVID-19 infection and sociodemographic factors on loneliness.						
**Outcome**	**Exposure**	**b**	**p-value**	**CI (lower)**	**CI (upper)**	**FDR corrected p-value**
Loneliness, June-July 2020	COVID-19 infection	0.261	0.326	-0.259	0.781	0.619
	Sex: Female (vs Male)	0.292	**0.000**	0.168	0.416	**0.000**
	COVID-19 infection*Female	0.214	0.365	-0.249	0.677	0.663
	Age group: 60-74 (vs 52-59)	-0.234	**0.011**	-0.416	-0.053	**0.055**
	Age group: 75+	-0.360	**0.001**	-0.580	-0.141	**0.008**
	COVID-19 infection*60-74	-0.461	0.180	-1.136	0.214	0.428
	COVID-19 infection*75+	-0.346	0.416	-1.179	0.488	0.700
	Work: Retired (vs employed)	0.115	0.164	-0.047	0.276	0.411
	Work: Other not working	0.305	0.066	-0.020	0.630	0.228
	COVID-19 infection*Retired	0.422	0.167	-0.177	1.020	0.413
	COVID-19 infection*Other not working	1.369	**0.015**	0.267	2.471	0.068
	Wealth: Medium (vs High)	-0.085	0.201	-0.216	0.045	0.451
	Wealth: Low	0.065	0.372	-0.078	0.208	0.670
	COVID-19 infection*Medium wealth	0.140	0.574	-0.348	0.629	0.829
	COVID-19 infection*Low wealth	-0.051	0.846	-0.567	0.465	0.978
	Living alone	0.619	**0.000**	0.461	0.777	**0.000**
	COVID-19 infection*Living alone	0.098	0.752	-0.510	0.707	0.938
Loneliness, Nov-Dec 2020	COVID-19 infection	-0.148	0.584	-0.677	0.382	0.834
	Sex: Female (vs Male)	0.234	**0.000**	0.106	0.363	**0.003**
	COVID-19 infection*Female	0.499	0.059	-0.019	1.018	0.209
	Age group: 60-74 (vs 52-59)	-0.209	**0.031**	-0.399	-0.019	0.125
	Age group: 75+	-0.340	**0.004**	-0.573	-0.107	**0.024**
	COVID-19 infection*60-74	-0.766	**0.024**	-1.430	-0.101	0.100
	COVID-19 infection*75+	-0.729	0.121	-1.649	0.191	0.339
	Work: Retired (vs employed)	0.147	0.085	-0.020	0.313	0.269
	Work: Other not working	0.329	**0.049**	0.001	0.657	0.184
	COVID-19 infection*Retired	0.481	0.107	-0.105	1.067	0.316
	COVID-19 infection*Other not working	0.757	0.109	-0.168	1.682	0.316
	Wealth: Medium (vs High)	-0.078	0.277	-0.219	0.063	0.573
	Wealth: Low	-0.037	0.639	-0.189	0.116	0.874
	COVID-19 infection*Medium wealth	0.411	0.123	-0.111	0.933	0.339
	COVID-19 infection*Low wealth	0.114	0.633	-0.355	0.583	0.870
	Living alone	0.582	**0.000**	0.416	0.748	**0.000**
	COVID-19 infection*Living alone	0.689	0.082	-0.087	1.464	0.264

Note. *Sample: ELSA COVID-19 Substudy (N=5,146); pooled estimates from linear/logistic regression models across 20 imputed datasets, weighted using survey weights; models adjusted for sex, age, pre-COVID-19 outcome, Nov-Dec 2020 COVID-19 infection (Nov-Dec 2020 outcome only), whether living alone, employment status, wealth, whether vulnerable to COVID-19, and limiting long-standing illness and including interaction effects between COVID-19 infection and sociodemographic factors; June-July 2020 = COVID-19 assessment 1; Nov-Dec 2020 = COVID-19 assessment 2; CI = 95% confidence interval; FDR = false discovery rate.*

**sTable8. Interaction effects between probable COVID-19 infection and sociodemographic factors on financial hardship – worried.**						
**Outcome**	**Exposure**	**OR**	**p-value**	**CI (lower)**	**CI (upper)**	**FDR corrected p-value**
Financial hardship – Worried, June-July 2020	COVID-19 infection	1.481	0.411	0.581	3.775	0.698
	Sex: Female (vs Male)	1.489	**0.001**	1.189	1.865	**0.004**
	COVID-19 infection*Female	0.948	0.887	0.457	1.967	0.978
	Age group: 60-74 (vs 52-59)	0.859	0.305	0.641	1.149	0.601
	Age group: 75+	0.470	**0.000**	0.309	0.714	**0.003**
	COVID-19 infection*60-74	0.590	0.294	0.220	1.582	0.598
	COVID-19 infection*75+	0.546	0.396	0.135	2.207	0.691
	Work: Retired (vs employed)	0.344	**0.000**	0.261	0.454	**0.000**
	Work: Other not working	0.971	0.895	0.630	1.498	0.978
	COVID-19 infection*Retired	2.772	**0.038**	1.057	7.272	0.151
	COVID-19 infection*Other not working	1.436	0.610	0.357	5.766	0.849
	Wealth: Medium (vs High)	1.448	**0.012**	1.084	1.935	0.058
	Wealth: Low	1.298	0.079	0.970	1.737	0.264
	COVID-19 infection*Medium wealth	0.435	0.054	0.187	1.013	0.195
	COVID-19 infection*Low wealth	1.179	0.694	0.521	2.668	0.900
	Living alone	1.226	0.124	0.946	1.588	0.340
	COVID-19 infection*Living alone	0.621	0.269	0.267	1.444	0.560
Financial hardship – Worried, Nov-Dec 2020	COVID-19 infection	1.351	0.555	0.497	3.668	0.819
	Sex: Female (vs Male)	1.436	**0.001**	1.149	1.794	**0.009**
	COVID-19 infection*Female	1.535	0.259	0.729	3.233	0.544
	Age group: 60-74 (vs 52-59)	0.770	0.078	0.576	1.030	0.263
	Age group: 75+	0.389	**0.000**	0.266	0.569	**0.000**
	COVID-19 infection*60-74	0.398	0.075	0.144	1.096	0.256
	COVID-19 infection*75+	0.601	0.489	0.142	2.539	0.772
	Work: Retired (vs employed)	0.446	**0.000**	0.338	0.588	**0.000**
	Work: Other not working	1.027	0.903	0.669	1.576	0.978
	COVID-19 infection*Retired	2.329	0.109	0.829	6.547	0.316
	COVID-19 infection*Other not working	0.848	0.800	0.237	3.034	0.967
	Wealth: Medium (vs High)	1.579	**0.003**	1.173	2.126	**0.015**
	Wealth: Low	1.521	**0.006**	1.130	2.047	**0.029**
	COVID-19 infection*Medium wealth	0.512	0.137	0.211	1.239	0.361
	COVID-19 infection*Low wealth	1.270	0.588	0.534	3.022	0.834
	Living alone	1.251	0.088	0.967	1.619	0.277
	COVID-19 infection*Living alone	0.490	0.108	0.206	1.168	0.316

Note. *Sample: ELSA COVID-19 Substudy (N=5,146); pooled estimates from linear/logistic regression models across 20 imputed datasets, weighted using survey weights; models adjusted for sex, age, pre-COVID-19 outcome, Nov-Dec 2020 COVID-19 infection (Nov-Dec 2020 outcome only), whether living alone, employment status, wealth, whether vulnerable to COVID-19, and limiting long-standing illness and including interaction effects between COVID-19 infection and sociodemographic factors; June-July 2020 = COVID-19 assessment 1; Nov-Dec 2020 = COVID-19 assessment 2; OR = odds ratio; CI = 95% confidence interval; FDR = false discovery rate.*

**sTable9. Interaction effects between probable COVID-19 infection and sociodemographic factors on financial hardship – worse off.**						
**Outcome**	**Exposure**	**OR**	**p-value**	**CI (lower)**	**(CI upper)**	**FDR corrected p-value**
Financial hardship – Worse off, June-July 2020	COVID-19 infection	1.699	0.197	0.759	3.803	0.449
	Sex: Female (vs Male)	0.788	**0.022**	0.643	0.967	0.097
	COVID-19 infection*Female	0.833	0.575	0.440	1.576	0.829
	Age group: 60-74 (vs 52-59)	0.891	0.436	0.668	1.190	0.722
	Age group: 75+	0.533	**0.001**	0.366	0.776	**0.007**
	COVID-19 infection*60-74	0.811	0.630	0.345	1.906	0.870
	COVID-19 infection*75+	1.185	0.765	0.389	3.605	0.947
	Work: Retired (vs employed)	0.477	**0.000**	0.366	0.620	**0.000**
	Work: Other not working	0.529	**0.005**	0.340	0.823	**0.025**
	COVID-19 infection*Retired	0.886	0.783	0.375	2.095	0.958
	COVID-19 infection*Other not working	1.296	0.645	0.430	3.900	0.874
	Wealth: Medium (vs High)	0.533	**0.000**	0.415	0.685	**0.000**
	Wealth: Low	0.377	**0.000**	0.287	0.496	**0.000**
	COVID-19 infection*Medium wealth	0.914	0.819	0.422	1.979	0.968
	COVID-19 infection*Low wealth	1.140	0.743	0.520	2.498	0.937
	Living alone	1.164	0.239	0.904	1.500	0.521
	COVID-19 infection*Living alone	0.972	0.946	0.431	2.193	0.980
Financial hardship – Worse off, Nov-Dec 2020	COVID-19 infection	1.083	0.850	0.472	2.485	0.978
	Sex: Female (vs Male)	0.826	0.081	0.666	1.024	0.264
	COVID-19 infection*Female	0.504	0.051	0.253	1.004	0.189
	Age group: 60-74 (vs 52-59)	0.693	**0.014**	0.518	0.928	0.064
	Age group: 75+	0.492	**0.000**	0.333	0.727	**0.003**
	COVID-19 infection*60-74	1.219	0.672	0.487	3.049	0.884
	COVID-19 infection*75+	1.528	0.506	0.438	5.324	0.785
	Work: Retired (vs employed)	0.497	**0.000**	0.377	0.656	**0.000**
	Work: Other not working	0.566	**0.009**	0.369	0.868	**0.046**
	COVID-19 infection*Retired	1.501	0.389	0.596	3.778	0.683
	COVID-19 infection*Other not working	3.566	**0.021**	1.215	10.469	0.091
	Wealth: Medium (vs High)	0.580	**0.000**	0.447	0.751	**0.000**
	Wealth: Low	0.389	**0.000**	0.293	0.517	**0.000**
	COVID-19 infection*Medium wealth	0.782	0.546	0.352	1.737	0.810
	COVID-19 infection*Low wealth	1.297	0.514	0.594	2.834	0.791
	Living alone	1.027	0.851	0.781	1.350	0.978
	COVID-19 infection*Living alone	0.662	0.328	0.290	1.512	0.619
Note. *Sample: ELSA COVID-19 Substudy (N=5,146); pooled estimates from linear/logistic regression models across 20 imputed datasets, weighted using survey weights; models adjusted for sex, age, pre-COVID-19 outcome, Nov-Dec 2020 COVID-19 infection (Nov-Dec 2020 outcome only), whether living alone, employment status, wealth, whether vulnerable to COVID-19, and limiting long-standing illness and including interaction effects between COVID-19 infection and sociodemographic factors; June-July 2020 = COVID-19 assessment 1; Nov-Dec 2020 = COVID-19 assessment 2; OR = odds ratio; CI = 95% confidence interval; FDR = false discovery rate.*						

sTable10. Interaction effects between probable COVID-19 infection and sociodemographic factors on infrequent real-time/written contact with family.						
**Outcome**	**Exposure**	**OR**	**p-value**	**CI (lower)**	**CI (upper)**	**FDR corrected p-value**
Infrequent real-time/written contact with family, June-July 2020	COVID-19 infection	0.275	0.172	0.043	1.753	0.418
	Sex: Female (vs Male)	0.448	**0.000**	0.328	0.611	**0.000**
	COVID-19 infection*Female	2.245	0.185	0.680	7.416	0.432
	Age group: 60-74 (vs 52-59)	1.042	0.873	0.632	1.717	0.978
	Age group: 75+	1.210	0.512	0.685	2.140	0.791
	COVID-19 infection*60-74	4.799	0.118	0.672	34.289	0.337
	COVID-19 infection*75+	2.343	0.434	0.278	19.759	0.722
	Work: Retired (vs employed)	0.972	0.904	0.609	1.550	0.978
	Work: Other not working	1.193	0.589	0.629	2.261	0.834
	COVID-19 infection*Retired	1.097	0.904	0.244	4.926	0.978
	COVID-19 infection*Other not working	NA				
	Wealth: Medium (vs High)	0.964	0.841	0.671	1.383	0.978
	Wealth: Low	0.866	0.484	0.580	1.295	0.772
	COVID-19 infection*Medium wealth	0.172	**0.015**	0.041	0.715	0.069
	COVID-19 infection*Low wealth	0.772	0.662	0.241	2.467	0.883
	Living alone	1.189	0.333	0.837	1.690	0.622
	COVID-19 infection*Living alone	1.403	0.589	0.411	4.786	0.834
Infrequent real-time/written contact with family, Nov-Dec 2020	COVID-19 infection	0.780	0.646	0.271	2.249	0.874
	Sex: Female (vs Male)	0.441	**0.000**	0.337	0.577	**0.000**
	COVID-19 infection*Female	1.148	0.761	0.472	2.790	0.946
	Age group: 60-74 (vs 52-59)	1.000	0.999	0.671	1.490	0.999
	Age group: 75+	1.176	0.490	0.743	1.861	0.772
	COVID-19 infection*60-74	1.077	0.908	0.304	3.812	0.978
	COVID-19 infection*75+	1.177	0.832	0.261	5.311	0.977
	Work: Retired (vs employed)	1.044	0.824	0.715	1.523	0.972
	Work: Other not working	1.417	0.211	0.821	2.447	0.464
	COVID-19 infection*Retired	1.455	0.560	0.413	5.129	0.822
	COVID-19 infection*Other not working	0.837	0.841	0.145	4.811	0.978
	Wealth: Medium (vs High)	0.770	0.092	0.568	1.044	0.285
	Wealth: Low	1.039	0.813	0.758	1.424	0.967
	COVID-19 infection*Medium wealth	1.035	0.947	0.367	2.921	0.980
	COVID-19 infection*Low wealth	0.940	0.900	0.358	2.469	0.978
	Living alone	1.173	0.305	0.865	1.592	0.601
	COVID-19 infection*Living alone	1.022	0.963	0.402	2.600	0.980

Note. *Sample: ELSA COVID-19 Substudy (N=5,146); pooled estimates from linear/logistic regression models across 20 imputed datasets, weighted using survey weights; models adjusted for sex, age, pre-COVID-19 outcome, Nov-Dec 2020 COVID-19 infection (Nov-Dec 2020 outcome only), whether living alone, employment status, wealth, whether vulnerable to COVID-19, and limiting long-standing illness and including interaction effects between COVID-19 infection and sociodemographic factors; June-July 2020 = COVID-19 assessment 1; Nov-Dec 2020 = COVID-19 assessment 2; OR = odds ratio; CI = 95% confidence interval; FDR = false discovery rate.*

**sTable11. Interaction effects between probable COVID-19 infection and sociodemographic factors on infrequent real-time/written contact with friends.**						
**Outcome**	**Exposure**	**OR**	**p-value**	**CI (lower)**	**CI (upper)**	**FDR corrected p-value**
Infrequent real-time/written contact with friends, June-July 2020	COVID-19 infection	0.395	0.126	0.120	1.299	0.340
	Sex: Female (vs Male)	0.428	**0.000**	0.332	0.553	**0.000**
	COVID-19 infection*Female	2.257	**0.047**	1.011	5.039	0.181
	Age group: 60-74 (vs 52-59)	0.990	0.958	0.680	1.440	0.980
	Age group: 75+	1.343	0.164	0.887	2.035	0.411
	COVID-19 infection*60-74	1.272	0.749	0.292	5.543	0.938
	COVID-19 infection*75+	1.441	0.676	0.259	8.001	0.884
	Work: Retired (vs employed)	0.866	0.383	0.626	1.197	0.678
	Work: Other not working	0.922	0.778	0.526	1.619	0.958
	COVID-19 infection*Retired	1.703	0.487	0.379	7.657	0.772
	COVID-19 infection*Other not working	0.790	0.803	0.124	5.035	0.967
	Wealth: Medium (vs High)	0.923	0.605	0.683	1.249	0.849
	Wealth: Low	0.975	0.878	0.706	1.346	0.978
	COVID-19 infection*Medium wealth	1.126	0.809	0.430	2.953	0.967
	COVID-19 infection*Low wealth	0.783	0.646	0.276	2.222	0.874
	Living alone	0.495	**0.000**	0.366	0.669	**0.000**
	COVID-19 infection*Living alone	0.971	0.955	0.340	2.770	0.980
Infrequent real-time/written contact with friends, Nov-Dec 2020	COVID-19 infection	1.438	0.461	0.548	3.777	0.752
	Sex: Female (vs Male)	0.423	**0.000**	0.337	0.531	**0.000**
	COVID-19 infection*Female	1.173	0.682	0.546	2.521	0.889
	Age group: 60-74 (vs 52-59)	1.018	0.914	0.731	1.420	0.979
	Age group: 75+	1.428	0.078	0.961	2.124	0.263
	COVID-19 infection*60-74	0.620	0.406	0.201	1.913	0.698
	COVID-19 infection*75+	1.100	0.890	0.284	4.260	0.978
	Work: Retired (vs employed)	0.909	0.537	0.672	1.230	0.807
	Work: Other not working	1.465	0.107	0.921	2.329	0.316
	COVID-19 infection*Retired	0.966	0.954	0.292	3.198	0.980
	COVID-19 infection*Other not working	1.047	0.945	0.280	3.915	0.980
	Wealth: Medium (vs High)	1.134	0.354	0.869	1.481	0.653
	Wealth: Low	1.147	0.333	0.869	1.512	0.622
	COVID-19 infection*Medium wealth	0.858	0.727	0.362	2.031	0.928
	COVID-19 infection*Low wealth	0.952	0.916	0.383	2.366	0.979
	Living alone	0.564	**0.000**	0.424	0.750	**0.001**
	COVID-19 infection*Living alone	1.134	0.787	0.455	2.826	0.960

Note. *Sample: ELSA COVID-19 Substudy (N=5,146); pooled estimates from linear/logistic regression models across 20 imputed datasets, weighted using survey weights; models adjusted for sex, age, pre-COVID-19 outcome, Nov-Dec 2020 COVID-19 infection (Nov-Dec 2020 outcome only), whether living alone, employment status, wealth, whether vulnerable to COVID-19, and limiting long-standing illness and including interaction effects between COVID-19 infection and sociodemographic factors; June-July 2020 = COVID-19 assessment 1; Nov-Dec 2020 = COVID-19 assessment 2; OR = odds ratio; CI = 95% confidence interval; FDR = false discovery rate.*

**sTable12. Interaction effects between probable COVID-19 infection and sociodemographic factors on infrequent real-time/written contact with family/friends (total score).**						
**Outcome**	**Exposure**	**b**	**p-value**	**CI (lower)**	**CI (upper)**	**FDR corrected p-value**
**Infrequent real-time/written contact with family/friends (total score), June-July 2020**	COVID-19 infection	0.395	0.600	-1.081	1.871	0.846
	Sex: Female (vs Male)	-1.842	**0.000**	-2.137	-1.547	**0.000**
	COVID-19 infection*Female	-0.266	0.608	-1.281	0.749	0.849
	Age group: 60-74 (vs 52-59)	0.031	0.901	-0.462	0.524	0.978
	Age group: 75+	1.508	**0.000**	0.931	2.086	**0.000**
	COVID-19 infection*60-74	0.028	0.972	-1.527	1.583	0.981
	COVID-19 infection*75+	-0.437	0.661	-2.392	1.518	0.883
	Work: Retired (vs employed)	0.296	0.166	-0.123	0.715	0.412
	Work: Other not working	0.579	0.096	-0.104	1.261	0.297
	COVID-19 infection*Retired	0.611	0.413	-0.853	2.075	0.699
	COVID-19 infection*Other not working	0.161	0.877	-1.880	2.202	0.978
	Wealth: Medium (vs High)	0.466	**0.010**	0.114	0.819	**0.047**
	Wealth: Low	0.462	**0.014**	0.093	0.832	0.066
	COVID-19 infection*Medium wealth	-0.974	0.131	-2.239	0.291	0.348
	COVID-19 infection*Low wealth	-0.665	0.323	-1.986	0.655	0.619
	Living alone	-0.872	**0.000**	-1.214	-0.530	**0.000**
	COVID-19 infection*Living alone	-0.559	0.364	-1.768	0.650	0.663
**Infrequent real-time/written contact with family/friends (total score), Nov-Dec 2020**	COVID-19 infection	0.167	0.813	-1.223	1.558	0.967
	Sex: Female (vs Male)	-1.880	**0.000**	-2.159	-1.601	**0.000**
	COVID-19 infection*Female	-0.023	0.962	-0.985	0.938	0.980
	Age group: 60-74 (vs 52-59)	-0.006	0.980	-0.473	0.461	0.986
	Age group: 75+	1.162	**0.000**	0.615	1.709	**0.000**
	COVID-19 infection*60-74	0.033	0.964	-1.439	1.506	0.980
	COVID-19 infection*75+	-0.071	0.940	-1.922	1.779	0.980
	Work: Retired (vs employed)	0.228	0.259	-0.168	0.625	0.544
	Work: Other not working	0.487	0.139	-0.159	1.133	0.363
	COVID-19 infection*Retired	-0.052	0.941	-1.439	1.335	0.980
	COVID-19 infection*Other not working	0.114	0.908	-1.816	2.044	0.978
	Wealth: Medium (vs High)	0.447	**0.009**	0.113	0.781	**0.044**
	Wealth: Low	0.176	0.325	-0.174	0.525	0.619
	COVID-19 infection*Medium wealth	-0.040	0.947	-1.215	1.135	0.980
	COVID-19 infection*Low wealth	-0.160	0.797	-1.386	1.065	0.967
	Living alone	-0.669	**0.000**	-0.992	-0.345	**0.001**
	COVID-19 infection*Living alone	-0.093	0.873	-1.239	1.052	0.978

Note. *Sample: ELSA COVID-19 Substudy (N=5,146); pooled estimates from linear/logistic regression models across 20 imputed datasets, weighted using survey weights; models adjusted for sex, age, pre-COVID-19 outcome, Nov-Dec 2020 COVID-19 infection (Nov-Dec 2020 outcome only), whether living alone, employment status, wealth, whether vulnerable to COVID-19, and limiting long-standing illness and including interaction effects between COVID-19 infection and sociodemographic factors; June-July 2020 = COVID-19 assessment 1 (June-July 2020); Nov-Dec 2020 = COVID-19 assessment 2 (Nov-Dec 2020); CI = 95% confidence interval; FDR = false discovery rate.*

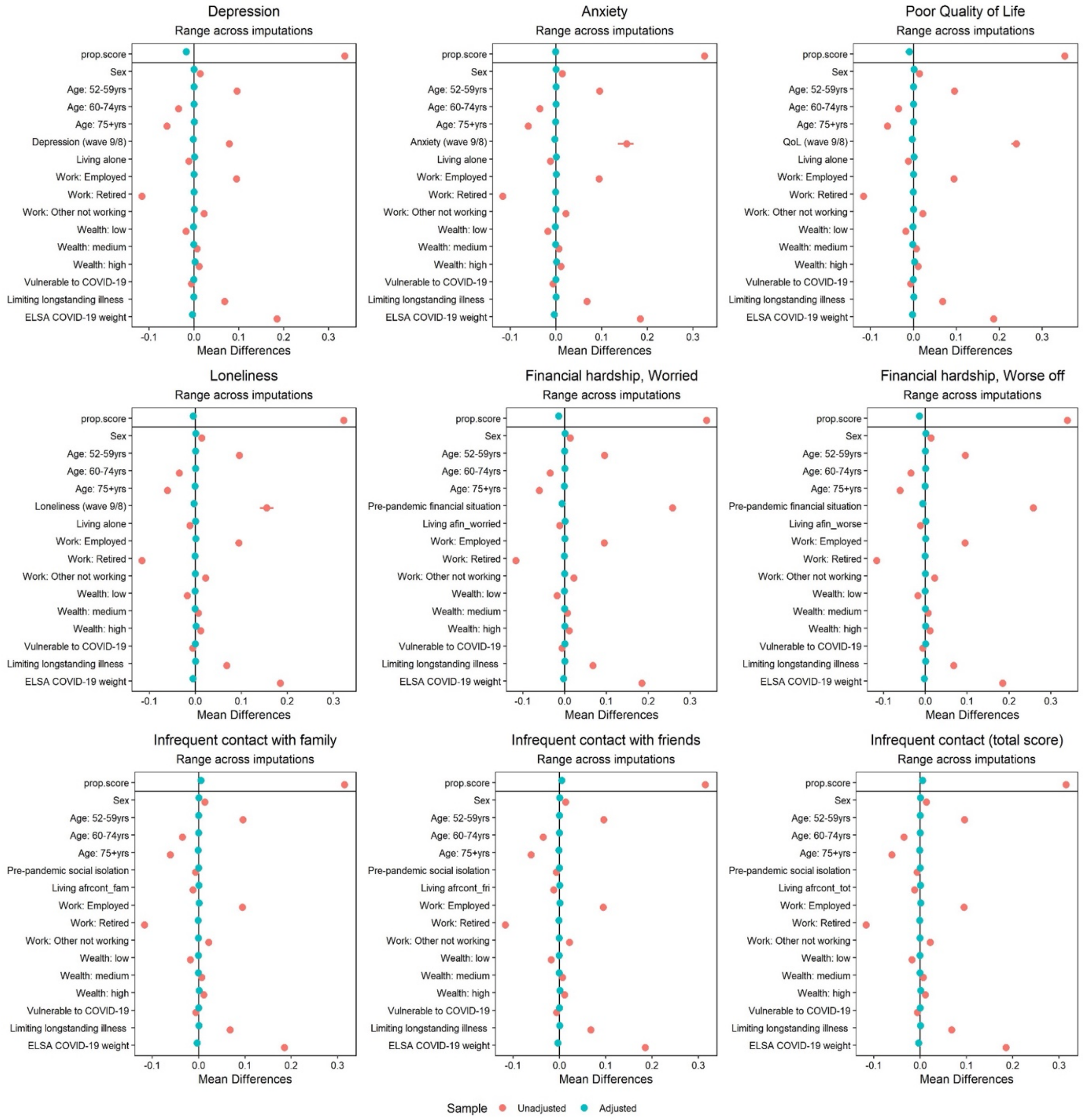

sFigure1. Balance of the covariates in the treatment and control groups across the imputed datasets before and after propensity score weighting.

***Note*.** *Standardised mean differences.*

sTable13. Associations between probable COVID-19 infection and mental health, financial hardship, and social connections (IPTW analyses).					
**Outcome**	**Exposure**	**OR/b**	**p-value**	**CI (lower)**	**CI (upper)**
Mental Health	COVID-19 infection (vs no infection)				
Depression, June-July 2020 (OR)		1.355	**0.010**	1.075	1.708
Depression, Nov-Dec 2020 (OR)		1.468	**0.000**	1.189	1.813
Anxiety, June-July 2020 (OR)		1.591	**0.003**	1.167	2.169
Anxiety, Nov-Dec 2020 (OR)		1.689	**0.000**	1.275	2.239
Poor QoL, June-July 2020 (b)		0.929	**0.008**	0.240	1.618
Poor QoL, Nov-Dec 2020 (b)		1.327	**0.000**	0.638	2.016
Loneliness, June-July 2020 (b)		0.382	**0.001**	0.154	0.609
Loneliness, Nov-Dec 2020 (b)		0.244	**0.039**	0.012	0.475
Financial Hardship					
Worried, June-July 2020 (OR)		1.216	0.079	0.977	1.514
Worried, Nov-Dec 2020 (OR)		1.145	0.234	0.916	1.431
Worse off, June-July 2020 (OR)		1.400	**0.005**	1.110	1.766
Worse off, Nov-Dec 2020 (OR)		1.166	0.225	0.910	1.493
Social Connections					
Infrequent contact with family, June-July 2020 (OR)		0.799	0.298	0.525	1.218
Infrequent contact with family, Nov-Dec 2020 (OR)		1.067	0.701	0.768	1.482
Infrequent contact with friends, June-July 2020 (OR)		0.974	0.872	0.704	1.347
Infrequent contact with friends, Nov-Dec 2020 (OR)		1.032	0.829	0.776	1.373
Infrequent contact with family/friends (total score), June-July 2020 (b)		-0.163	0.555	-0.705	0.379
Infrequent contact with family/friends (total score), Nov-Dec 2020 (b)		-0.003	0.991	-0.506	0.500

Note. *ELSA COVID-19 longitudinal sample (N=5146); pooled estimates across 20 imputed datasets; logistic/linear regression models weighted using propensity scores including sex, age, pre-COVID-19 outcomes, whether living alone, employment status, wealth, whether vulnerable to COVID-19, limiting long-standing illness, and the survey weights as predictors of the probability of probable COVID-19 infection; June-July 2020 = COVID-19 assessment 1; Nov-Dec 2020 = COVID-19 assessment 2; OR = odds ratio; CI = 95% confidence interval; QoL = quality of life; IPTW = inverse probability of treatment weighting.*

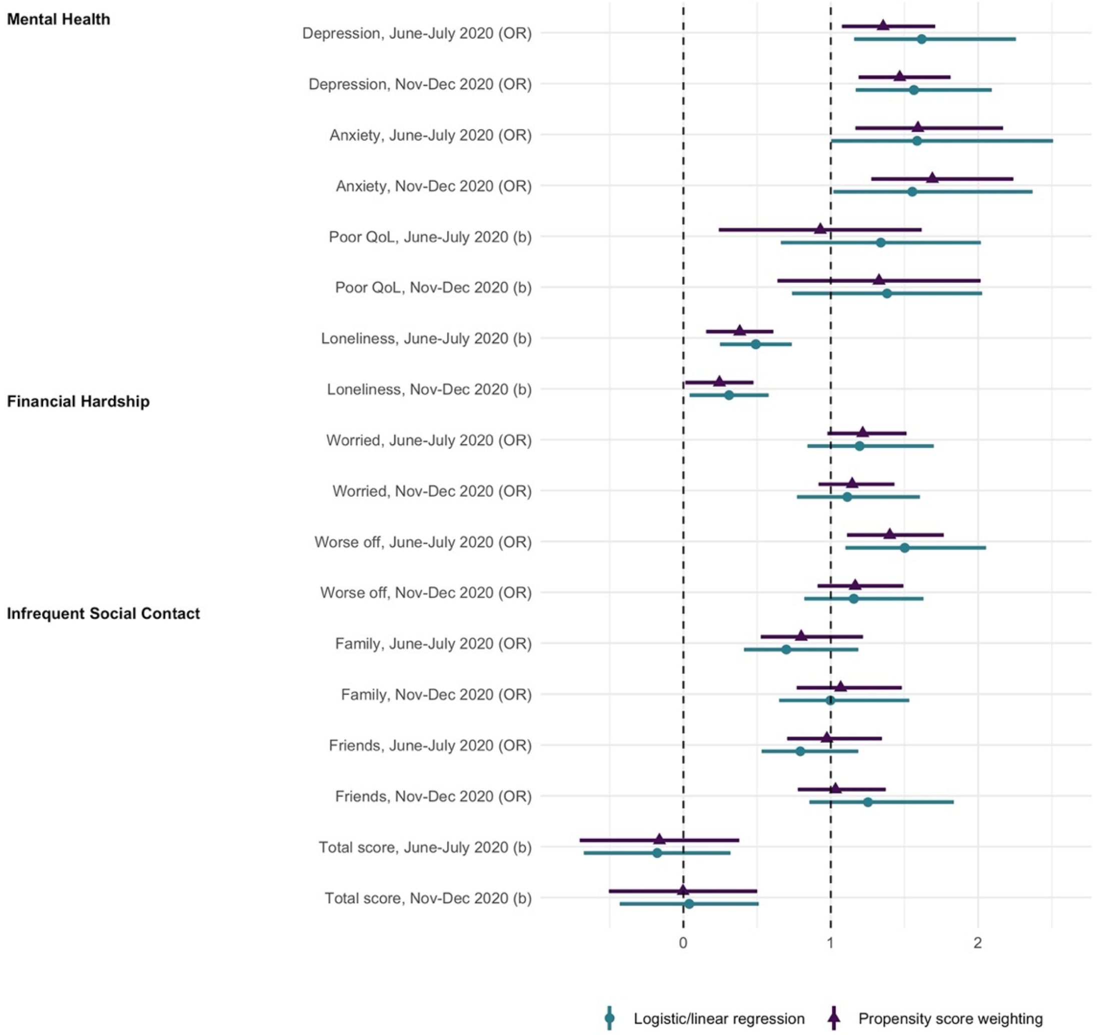

sFigure2. Associations of probable COVID-19 infection with mental health, financial hardship, and social connections (95% confidence intervals).

Note. *ELSA COVID-19 longitudinal sample (N=5146); pooled estimates across 20 imputed datasets; Logistic/linear regression models: adjusted for sex, age, pre-COVID-19 outcomes, whether living alone, employment status, wealth, whether vulnerable to COVID-19, Nov-Dec 2020 COVID-19 infection (Nov-Dec 2020 outcome only), and limiting long-standing illness, and weighted using survey weights; Propensity score weighting: logistic/linear regression models weighted using propensity scores with sex, age, pre-COVID-19 outcomes, whether living alone, employment status, wealth, whether vulnerable to COVID-19, limiting long-standing illness, and survey weights as predictors of the probability of probable COVID-19 infection; June-July 2020 = COVID-19 assessment 1; Nov-Dec 2020 = COVID-19 assessment 2; QoL = quality of life.*

sTable14. Associations of probable COVID-19 infection (definitions 2 and 3) with mental health, financial hardship, and social connections.

Outcome	Exposure	OR/b	p-value	CI (lower)	CI (upper)
Depression, June-July 2020	COVID-19 infection, def 2	1.337	0.249	0.816	2.190
	COVID-19 infection, def 3	1.436	0.059	0.987	2.090
Depression, Nov-Dec 2020	COVID-19 infection, def 2	1.337	0.189	0.867	2.062
	COVID-19 infection, def 3	1.430	**0.031**	1.033	1.980
Anxiety, June-July 2020	COVID-19 infection, def 2	1.695	0.155	0.818	3.511
	COVID-19 infection, def 3	1.272	0.352	0.766	2.111
Anxiety, Nov-Dec 2020	COVID-19 infection, def 2	1.728	0.095	0.909	3.286
	COVID-19 infection, def 3	1.392	0.146	0.891	2.175
Poor QoL, June-July 2020	COVID-19 infection, def 2	1.182	**0.018**	0.205	2.159
	COVID-19 infection, def 3	1.139	**0.001**	0.496	1.782
Poor QoL, Nov-Dec 2020	COVID-19 infection, def 2	0.911	0.088	-0.134	1.956
	COVID-19 infection, def 3	1.203	**0.001**	0.490	1.917
Loneliness, June-July 2020	COVID-19 infection, def 2	0.551	**0.002**	0.204	0.898
	COVID-19 infection, def 3	0.524	**0.000**	0.260	0.789
Loneliness, Nov-Dec 2020	COVID-19 infection, def 2	0.380	0.139	-0.123	0.884
	COVID-19 infection, def 3	0.283	0.069	-0.022	0.588
Financial hardship – Worried, June-July 2020	COVID-19 infection, def 2	1.165	0.641	0.614	2.210
	COVID-19 infection, def 3	1.331	0.151	0.901	1.967
Financial hardship – Worried, Nov-Dec 2020	COVID-19 infection, def 2	1.351	0.336	0.732	2.494
	COVID-19 infection, def 3	1.175	0.441	0.780	1.770
Financial hardship – Worse off, June-July 2020	COVID-19 infection, def 2	1.756	**0.028**	1.062	2.901
	COVID-19 infection, def 3	1.526	**0.017**	1.077	2.162
Financial hardship – Worse off, Nov-Dec 2020	COVID-19 infection, def 2	1.286	0.364	0.747	2.216
	COVID-19 infection, def 3	1.186	0.380	0.810	1.735
Infrequent contact with family, June-July 2020	COVID-19 infection, def 2	0.450	0.065	0.193	1.051
	COVID-19 infection, def 3	0.605	0.095	0.335	1.091
Infrequent contact with family, Nov-Dec 2020	COVID-19 infection, def 2	0.836	0.626	0.407	1.717
	COVID-19 infection, def 3	0.922	0.737	0.573	1.483
Infrequent contact with friends, June-July 2020	COVID-19 infection, def 2	0.677	0.268	0.340	1.350
	COVID-19 infection, def 3	0.806	0.342	0.517	1.258
Infrequent contact with friends, Nov-Dec 2020	COVID-19 infection, def 2	0.870	0.720	0.408	1.858
	COVID-19 infection, def 3	1.129	0.589	0.727	1.752
Infrequent contact with family/friends (total score), June-July 2020	COVID-19 infection, def 2	-0.199	0.638	-1.028	0.630
	COVID-19 infection, def 3	-0.258	0.358	-0.807	0.291
Infrequent contact with family/friends (total score), Nov-Dec 2020	COVID-19 infection, def 2	-0.131	0.746	-0.919	0.658
	COVID-19 infection, def 3	-0.022	0.935	-0.543	0.499

***Note.** Sample: ELSA COVID-19 Substudy (N=5,146); pooled estimates from linear/logistic regression models across 20 imputed datasets, weighted using survey weights; Models adjusted for sex, age groups, pre-COVID-19 outcome, Nov-Dec 2020 COVID-19 infection (Nov-Dec 2020 outcome only), whether living alone, employment status, wealth, whether vulnerable to COVID-19, limiting long-standing illness; June-July 2020 = COVID-19 assessment 1; Nov-Dec 2020 = COVID-19 assessment 2; OR = odds ratio; CI = 95% confidence interval; QoL = quality of life; COVID-19 infection, def 2 = Positive COVID-19 test result, or hospitalisation due to COVID-19, or at least two of the three core symptoms as defined by NHS England (i.e. high temperature, new continuous cough, and loss of sense of smell/taste); COVID-19 infection, def 3 = Positive COVID-19 test result, or hospitalisation due to COVID-19, or a new continuous cough, or loss of sense of smell or taste.*

**sTable15. Associations of probable COVID-19 infection with real-time contact with family.**					
**Outcome**	**Exposure**	**OR**	**p-value**	**CI (lower)**	**CI (upper)**
a) Overall					
Infrequent real-time contact with family, June-July 2020	COVID-19 infection	0.978	0.913	0.659	1.451
Infrequent real-time contact with family, Nov-Dec 2020	COVID-19 infection	0.890	0.529	0.618	1.280
b) Interaction effects with sociodemographic factors					
Infrequent real-time contact with family, June-July 2020	COVID-19 infection	1.435	0.432	0.583	3.533
	Sex: Female (vs Male)	0.598	**0.000**	0.471	0.759
	COVID-19 infection*Female	1.113	0.797	0.493	2.515
	Age group: 60-74 (vs 52-59)	0.851	0.393	0.587	1.233
	Age group: 75+	0.709	0.120	0.460	1.093
	COVID-19 infection*60-74	1.363	0.560	0.481	3.865
	COVID-19 infection*75+	1.103	0.886	0.289	4.205
	Work: Retired (vs employed)	1.144	0.449	0.807	1.622
	Work: Other not working	1.339	0.225	0.835	2.147
	COVID-19 infection*Retired	0.689	0.495	0.236	2.008
	COVID-19 infection*Other not working	0.764	0.727	0.168	3.479
	Wealth: Medium (vs High)	0.868	0.318	0.657	1.146
	Wealth: Low	0.911	0.539	0.677	1.226
	COVID-19 infection*Medium wealth	0.405	**0.047**	0.166	0.990
	COVID-19 infection*Low wealth	0.654	0.342	0.272	1.572
	Living alone	1.113	0.447	0.844	1.469
	COVID-19 infection*Living alone	1.039	0.934	0.421	2.561
Infrequent real-time contact with family, Nov-Dec 2020	COVID-19 infection	0.631	0.294	0.267	1.491
	Sex: Female (vs Male)	0.492	**0.000**	0.396	0.613
	COVID-19 infection*Female	1.323	0.445	0.645	2.715
	Age group: 60-74 (vs 52-59)	1.147	0.407	0.829	1.589
	Age group: 75+	0.906	0.610	0.620	1.324
	COVID-19 infection*60-74	0.685	0.470	0.245	1.913
	COVID-19 infection*75+	0.800	0.730	0.226	2.833
	Work: Retired (vs employed)	0.951	0.739	0.706	1.280
	Work: Other not working	1.458	0.101	0.929	2.288
	COVID-19 infection*Retired	2.236	0.120	0.811	6.165
	COVID-19 infection*Other not working	0.915	0.898	0.235	3.566
	Wealth: Medium (vs High)	0.936	0.601	0.729	1.201
	Wealth: Low	1.039	0.768	0.804	1.344
	COVID-19 infection*Medium wealth	1.679	0.224	0.729	3.866
	COVID-19 infection*Low wealth	1.071	0.876	0.452	2.540
	Living alone	1.208	0.137	0.942	1.550
	COVID-19 infection*Living alone	0.734	0.480	0.311	1.733

Note. *Sample: ELSA COVID-19 Substudy (N=5,146); pooled estimates from logistic regression models across 20 imputed datasets, weighted using survey weights; Models adjusted for sex, age groups, pre-COVID-19 outcome, Nov-Dec 2020 COVID-19 infection (Nov-Dec 2020 outcome only), whether living alone, employment status, wealth, whether vulnerable to COVID-19, limiting long-standing illness; June-July 2020 = COVID-19 assessment 1; Nov-Dec 2020 = COVID-19 assessment 2; OR = odds ratio; CI = 95% confidence interval.*

sTable16. Associations of probable COVID-19 infection with real-time contact with friends.					
**Outcome**	**Exposure**	**OR**	**p-value**	**CI (lower)**	**CI (upper)**
a) Overall					
Infrequent real-time contact with friends, June-July 2020	COVID-19 infection	0.861	0.393	0.611	1.213
Infrequent real-time contact with friends, Nov-Dec 2020	COVID-19 infection	1.171	0.323	0.856	1.600
b) Interaction effects with sociodemographic factors					
Infrequent real-time contact with friends, June-July 2020	COVID-19 infection	0.446	0.066	0.189	1.055
	Sex: Female (vs Male)	0.573	**0.000**	0.469	0.701
	COVID-19 infection*Female	1.989	**0.044**	1.019	3.882
	Age group: 60-74 (vs 52-59)	1.013	0.930	0.752	1.365
	Age group: 75+	1.010	0.955	0.712	1.433
	COVID-19 infection*60-74	0.924	0.885	0.317	2.691
	COVID-19 infection*75+	0.785	0.712	0.217	2.839
	Work: Retired (vs employed)	0.688	**0.006**	0.527	0.897
	Work: Other not working	0.796	0.303	0.516	1.229
	COVID-19 infection*Retired	2.482	0.091	0.865	7.122
	COVID-19 infection*Other not working	2.380	0.196	0.639	8.858
	Wealth: Medium (vs High)	0.965	0.769	0.759	1.226
	Wealth: Low	1.019	0.886	0.793	1.309
	COVID-19 infection*Medium wealth	0.940	0.874	0.439	2.013
	COVID-19 infection*Low wealth	0.679	0.337	0.308	1.496
	Living alone	0.556	**0.000**	0.432	0.717
	COVID-19 infection*Living alone	1.652	0.223	0.737	3.701
Infrequent real-time contact with friends, Nov-Dec 2020	COVID-19 infection	0.754	0.471	0.349	1.626
	Sex: Female (vs Male)	0.562	**0.000**	0.468	0.674
	COVID-19 infection*Female	1.332	0.355	0.725	2.447
	Age group: 60-74 (vs 52-59)	0.880	0.336	0.678	1.142
	Age group: 75+	0.870	0.387	0.634	1.193
	COVID-19 infection*60-74	0.661	0.348	0.279	1.568
	COVID-19 infection*75+	0.943	0.915	0.320	2.778
	Work: Retired (vs employed)	0.748	**0.016**	0.591	0.947
	Work: Other not working	1.217	0.320	0.827	1.790
	COVID-19 infection*Retired	1.474	0.392	0.606	3.588
	COVID-19 infection*Other not working	0.793	0.682	0.262	2.405
	Wealth: Medium (vs High)	1.054	0.623	0.855	1.298
	Wealth: Low	0.895	0.337	0.714	1.122
	COVID-19 infection*Medium wealth	1.257	0.532	0.613	2.577
	COVID-19 infection*Low wealth	1.416	0.363	0.669	2.998
	Living alone	0.532	**0.000**	0.421	0.671
	COVID-19 infection*Living alone	1.649	0.183	0.790	3.443

Note. *Sample: ELSA COVID-19 Substudy (N=5,146); pooled estimates from logistic regression models across 20 imputed datasets, weighted using survey weights; Models adjusted for sex, age groups, pre-COVID-19 outcome, Nov-Dec 2020 COVID-19 infection (Nov-Dec 2020 outcome only), whether living alone, employment status, wealth, whether vulnerable to COVID-19, limiting long-standing illness; June-July 2020 = COVID-19 assessment 1; Nov-Dec 2020 = COVID-19 assessment 2; OR = odds ratio; CI = 95% confidence interval.*

**sTable17. Associations of probable COVID-19 infection with mental health, financial hardship, and social connections – Complete-case analyses.**						
**Outcome**	**Exposure**	**OR/b**	**p-value**	**CI (lower)**	**CI (upper)**	**N**
Depression, June-July 2020 (OR)	COVID-19 infection (vs no infection)	1.489	**0.026**	1.048	2.116	5016
Depression, Nov-Dec 2020 (OR)		1.650	**0.001**	1.225	2.224	5017
Anxiety, June-July 2020 (OR)		1.616	**0.047**	1.007	2.593	4855
Anxiety, Nov-Dec 2020 (OR)		1.634	**0.023**	1.069	2.498	4854
Poor QoL, June-July 2020 (b)		1.197	**0.001**	0.505	1.890	4898
Poor QoL, Nov-Dec 2020 (b)		1.291	**0.000**	0.643	1.940	4899
Loneliness, June-July 2020 (b)		0.495	**0.000**	0.242	0.747	4894
Loneliness, Nov-Dec 2020 (b)		0.286	**0.050**	0.000	0.571	4897
Financial Hardship: Worried, June-July 2020 (OR)		1.247	0.224	0.874	1.780	5069
Financial Hardship: Worried, Nov-Dec 2020 (OR)		1.119	0.558	0.769	1.628	5070
Financial Hardship: Worse off, June-July 2020 (OR)		1.530	**0.008**	1.115	2.099	5069
Financial Hardship: Worse off, Nov-Dec 2020 (OR)		1.219	0.261	0.863	1.720	5068
Infrequent contact: Family, June-July 2020 (OR)		0.794	0.413	0.457	1.379	4892
Infrequent contact: Family, Nov-Dec 2020 (OR)		1.109	0.646	0.714	1.722	4898
Infrequent contact: Friends, June-July 2020 (OR)		0.864	0.491	0.570	1.310	4901
Infrequent contact: Friends, Nov-Dec 2020 (OR)		1.372	0.116	0.924	2.037	4902
Infrequent contact: Total, June-July 2020 (b)		-0.209	0.597	-0.987	0.568	4902
Infrequent contact: Total, Nov-Dec 2020 (b)		0.108	0.767	-0.608	0.824	4902

Note. *Sample: ELSA COVID-19 Substudy (N=5,146); estimates from linear/logistic regression models, weighted using survey weights; Models adjusted for sex, age groups, pre-COVID-19 outcome, Nov-Dec 2020 COVID-19 infection (Nov-Dec 2020 outcome only), whether living alone, employment status, wealth, whether vulnerable to COVID-19, limiting long-standing illness; June-July 2020 = COVID-19 assessment 1; Nov-Dec 2020 = COVID-19 assessment 2; OR = odds ratio; CI = 95% confidence interval; QoL = quality of life.*

**sTable18. Associations between probable COVID-19 infection and Depression – Adjustment for and interactions with physical and mental health conditions.**						
**Outcome**	**Exposure**	**Model**	**OR**	**p-value**	**CI (lower)**	**CI (upper)**
a) Overall						
Depression, COVID-19 w1	COVID-19 infection	1	1.617	**0.005**	1.158	2.258
	COVID-19 infection	2	1.533	**0.011**	1.105	2.125
Depression, COVID-19 w2	COVID-19 infection	1	1.564	**0.003**	1.169	2.092
	COVID-19 infection	2	1.522	**0.004**	1.139	2.033
b) Interaction effects with mental and physical health conditions						
Depression, COVID-19 w1	COVID-19 infection	3	1.037	0.944	0.381	2.821
	Mental health condition	3	2.306	**0.000**	1.620	3.283
	COVID-19 infection*Mental health condition	3	2.513	0.069	0.931	6.781
	New physical health condition	3	1.455	**0.005**	1.118	1.893
	COVID-19 infection*New physical health condition	3	0.519	0.065	0.259	1.043
Depression, COVID-19 w2	COVID-19 infection	3	1.519	0.345	0.637	3.622
	Mental health condition	3	2.196	**0.000**	1.583	3.046
	COVID-19 infection*Mental health condition	3	2.281	0.089	0.882	5.899
	New physical health condition	3	1.319	**0.012**	1.062	1.638
	COVID-19 infection*New physical health condition	3	0.827	0.564	0.435	1.575

Note. *Sample: ELSA COVID-19 Substudy (N=5,146). Weighted pooled estimates from logistic regression models across 20 imputed datasets. Model 1: adjusted for sex, age groups, pre-COVID-19 depression, w2 COVID-19 infection (w2 outcome only), whether living alone, employment status, wealth, whether vulnerable to COVID-19, limiting long-standing illness; Model 2: Model 1 + mental and physical health conditions; Model 3: Model 2 + interaction effects of COVID-19 infection with sex, age, employment status, wealth, whether living alone, mental health condition, and new physical health condition. OR = odds ratio. CI = 95% confidence interval. COVID-19 infection, def 1 = Positive COVID-19 test result; or hospitalisation due to COVID-19; or at least one of the three core symptoms as defined by NHS England (i.e. high temperature, new continuous cough, and loss of sense of smell/taste).*

**sTable19. Associations between probable COVID-19 infection and Anxiety – Adjustment for and interactions with physical and mental health conditions.**						
**Outcome**	**Exposure**	**Model**	**OR**	**p-value**	**CI (lower)**	**CI (upper)**
a) Overall						
Anxiety, COVID-19 w1	COVID-19 infection	1	1.586	**0.049**	1.003	2.508
	COVID-19 infection	2	1.519	0.077	0.955	2.415
Anxiety, COVID-19 w2	COVID-19 infection	1	1.553	**0.041**	1.018	2.370
	COVID-19 infection	2	1.465	0.079	0.956	2.243
b) Interaction effects with mental and physical health conditions						
Anxiety, COVID-19 w1	COVID-19 infection	3	0.578	0.460	0.135	2.476
	Mental health condition	3	1.472	0.096	0.934	2.322
	COVID-19 infection*Mental health condition	3	1.035	0.954	0.322	3.326
	New physical health condition	3	1.433	**0.049**	1.002	2.050
	COVID-19 infection*New physical health condition	3	0.543	0.198	0.215	1.375
Anxiety, COVID-19 w2	COVID-19 infection	3	0.263	0.085	0.058	1.201
	Mental health condition	3	0.288	0.072	0.074	1.118
	COVID-19 infection*Mental health condition	3	1.580	0.248	0.726	3.439
	New physical health condition	3	1.462	**0.026**	1.046	2.042
	COVID-19 infection*New physical health condition	3	1.580	0.248	0.726	3.439

Note. *Sample: ELSA COVID-19 Substudy (N=5,146). Weighted pooled estimates from logistic regression models across 20 imputed datasets. Model 1: adjusted for sex, age groups, pre-COVID-19 anxiety, w2 COVID-19 infection (w2 outcome only), whether living alone, employment status, wealth, whether vulnerable to COVID-19, limiting long-standing illness; Model 2: Model 1 + mental and physical health conditions; Model 3: Model 2 + interaction effects of COVID-19 infection with sex, age, employment status, wealth, whether living alone, mental health condition, and new physical health condition. OR = odds ratio. CI = 95% confidence interval. COVID-19 infection, def 1 = Positive COVID-19 test result; or hospitalisation due to COVID-19; or at least one of the three core symptoms as defined by NHS England (i.e. high temperature, new continuous cough, and loss of sense of smell/taste).*

sTable20. Associations between probable COVID-19 infection and Poor Quality of Life (QoL) – Adjustment for and interactions with physical and mental health conditions.

**Outcome**	**Exposure**	**Model**	**b**	**p-value**	**CI (lower)**	**CI (upper)**
a) Overall						
Poor QoL, COVID-19 w1	COVID-19 infection	1	1.340	**0.000**	0.661	2.018
	COVID-19 infection	2	1.215	**0.000**	0.542	1.889
Poor QoL, COVID-19 w2	COVID-19 infection	1	1.382	**0.000**	0.737	2.027
	COVID-19 infection	2	1.270	**0.000**	0.624	1.916
b) Interaction effects with mental and physical health conditions						
Poor QoL, COVID-19 w1	COVID-19 infection	3	0.755	0.288	-0.639	2.149
	Mental health condition	3	0.616	0.135	-0.192	1.424
	COVID-19 infection*Mental health condition	3	0.214	0.854	-2.063	2.490
	New physical health condition	3	1.187	**0.000**	0.736	1.638
	COVID-19 infection*New physical health condition	3	-0.062	0.925	-1.341	1.218
Poor QoL, COVID-19 w2	COVID-19 infection	3	0.857	0.297	-0.754	2.469
	Mental health condition	3	0.901	**0.013**	0.192	1.610
	COVID-19 infection*Mental health condition	3	-0.586	0.592	-2.728	1.556
	New physical health condition	3	1.109	**0.000**	0.667	1.551
	COVID-19 infection*New physical health condition	3	-0.019	0.977	-1.300	1.263

Note. *Sample: ELSA COVID-19 Substudy (N=5,146). Weighted pooled estimates from linear regression models across 20 imputed datasets. Model 1: adjusted for sex, age groups, pre-COVID-19 QoL, w2 COVID-19 infection (w2 outcome only), whether living alone, employment status, wealth, whether vulnerable to COVID-19, limiting long-standing illness; Model 2: Model 1 + mental and physical health conditions; Model 3: Model 2 + interaction effects of COVID-19 infection with sex, age, employment status, wealth, whether living alone, mental health condition, and new physical health condition. QoL = quality of life. CI = 95% confidence interval. COVID-19 infection, def 1 = Positive COVID-19 test result; or hospitalisation due to COVID-19; or at least one of the three core symptoms as defined by NHS England (i.e. high temperature, new continuous cough, and loss of sense of smell/taste).*

sTable21. Associations between probable COVID-19 infection and Loneliness – Adjustment for and interactions with physical and mental health conditions.

Outcome	Exposure	Model	b	p-value	CI (lower)	CI (upper)
a) Overall						
Loneliness, COVID-19 w1	COVID-19 infection	1	0.491	**0.000**	0.247	0.735
	COVID-19 infection	2	0.467	**0.000**	0.222	0.711
Loneliness, COVID-19 w2	COVID-19 infection	1	0.310	**0.024**	0.042	0.577
	COVID-19 infection	2	0.295	**0.033**	0.024	0.566
b) Interaction effects with mental and physical health conditions						
Loneliness, COVID-19 w1	COVID-19 infection	3	0.368	0.183	-0.173	0.910
	Mental health condition	3	0.129	0.349	-0.141	0.399
	COVID-19 infection*Mental health condition	3	0.730	0.098	-0.134	1.595
	New physical health condition	3	0.291	**0.000**	0.151	0.431
	COVID-19 infection*New physical health condition	3	-0.524	**0.031**	-1.002	-0.047
Loneliness, COVID-19 w2	COVID-19 infection	3	0.010	0.973	-0.545	0.565
	Mental health condition	3	0.212	0.192	-0.107	0.530
	COVID-19 infection*Mental health condition	3	0.247	0.596	-0.666	1.159
	New physical health condition	3	0.212	**0.006**	0.062	0.362
	COVID-19 infection*New physical health condition	3	-0.741	**0.002**	-1.212	-0.270

Note. *Sample: ELSA COVID-19 Substudy (N=5,146). Weighted pooled estimates from linear regression models across 20 imputed datasets. Model 1: adjusted for sex, age groups, pre-COVID-19 loneliness, w2 COVID-19 infection (w2 outcome only), whether living alone, employment status, wealth, whether vulnerable to COVID-19, limiting long-standing illness; Model 2: Model 1 + mental and physical health conditions; Model 3: Model 2 + interaction effects of COVID-19 infection with sex, age, employment status, wealth, whether living alone, mental health condition, and new physical health condition. CI = 95% confidence interval. COVID-19 infection, def 1 = Positive COVID-19 test result; or hospitalisation due to COVID-19; or at least one of the three core symptoms as defined by NHS England (i.e. high temperature, new continuous cough, and loss of sense of smell/taste).*

**sTable22. Associations between probable COVID-19 infection and Worries about future financial situation – Adjustment for and interactions with physical and mental health conditions.**						
**Outcome**	**Exposure**	**Model**	**OR**	**p-value**	**CI (lower)**	**CI (upper)**
a) Overall						
Worried, COVID-19 w1	COVID-19 infection	1	1.196	0.318	0.842	1.699
	COVID-19 infection	2	1.182	0.357	0.829	1.685
Worried, COVID-19 w2	COVID-19 infection	1	1.112	0.571	0.770	1.606
	COVID-19 infection	2	1.069	0.721	0.741	1.542
b) Interaction effects with mental and physical health conditions						
Worried, COVID-19 w1	COVID-19 infection	3	1.410	0.491	0.531	3.747
	Mental health condition	3	1.381	0.106	0.934	2.043
	COVID-19 infection*Mental health condition	3	0.994	0.991	0.319	3.094
	New physical health condition	3	0.962	0.793	0.722	1.282
	COVID-19 infection*New physical health condition	3	1.390	0.390	0.656	2.946
Worried, COVID-19 w2	COVID-19 infection	3	1.229	0.711	0.412	3.673
	Mental health condition	3	1.553	**0.028**	1.050	2.296
	COVID-19 infection*Mental health condition	3	1.395	0.537	0.484	4.019
	New physical health condition	3	1.253	0.081	0.973	1.615
	COVID-19 infection*New physical health condition	3	1.297	0.520	0.588	2.859

Note. *Sample: ELSA COVID-19 Substudy (N=5,146). Weighted pooled estimates from logistic regression models across 20 imputed datasets. Model 1: adjusted for sex, age groups, pre-COVID-19 financial situation, w2 COVID-19 infection (w2 outcome only), whether living alone, employment status, wealth, whether vulnerable to COVID-19, limiting long-standing illness; Model 2: Model 1 + mental and physical health conditions; Model 3: Model 2 + interaction effects of COVID-19 infection with sex, age, employment status, wealth, whether living alone, mental health condition, and new physical health condition. OR = odds ratio. CI = 95% confidence interval. COVID-19 infection, def 1 = Positive COVID-19 test result; or hospitalisation due to COVID-19; or at least one of the three core symptoms as defined by NHS England (i.e. high temperature, new continuous cough, and loss of sense of smell/taste).*

**sTable23. Associations between probable COVID-19 infection and Current financial situation compared with pre-COVID-19 – Adjustment for and interactions with physical and mental health conditions.**						
**Outcome**	**Exposure**	**Model**	**OR**	**p-value**	**CI (lower)**	**CI (upper)**
a) Overall						
Worse off, COVID-19 w1	COVID-19 infection	1	1.502	**0.011**	1.099	2.053
	COVID-19 infection	2	1.496	**0.012**	1.093	2.047
Worse off, COVID-19 w2	COVID-19 infection	1	1.156	0.407	0.821	1.629
	COVID-19 infection	2	1.151	0.420	0.818	1.620
b) Interaction effects with mental and physical health conditions						
Worse off, COVID-19 w1	COVID-19 infection	3	1.742	0.182	0.771	3.936
	Mental health condition	3	1.191	0.382	0.805	1.763
	COVID-19 infection*Mental health condition	3	0.684	0.462	0.248	1.883
	New physical health condition	3	1.054	0.675	0.824	1.350
	COVID-19 infection*New physical health condition	3	0.860	0.671	0.430	1.722
Worse off, COVID-19 w2	COVID-19 infection	3	0.995	0.991	0.430	2.302
	Mental health condition	3	1.014	0.946	0.669	1.538
	COVID-19 infection*Mental health condition	3	0.434	0.155	0.138	1.370
	New physical health condition	3	1.007	0.961	0.770	1.316
	COVID-19 infection*New physical health condition	3	1.393	0.378	0.667	2.907

Note. *Sample: ELSA COVID-19 Substudy (N=5,146). Weighted pooled estimates from logistic regression models across 20 imputed datasets. Model 1: adjusted for sex, age groups, pre-COVID-19 financial situation, w2 COVID-19 infection (w2 outcome only), whether living alone, employment status, wealth, whether vulnerable to COVID-19, limiting long-standing illness; Model 2: Model 1 + mental and physical health conditions; Model 3: Model 2 + interaction effects of COVID-19 infection with sex, age, employment status, wealth, whether living alone, mental health condition, and new physical health condition. OR = odds ratio. CI = 95% confidence interval. COVID-19 infection, def 1 = Positive COVID-19 test result; or hospitalisation due to COVID-19; or at least one of the three core symptoms as defined by NHS England (i.e. high temperature, new continuous cough, and loss of sense of smell/taste).*

sTable24. Associations between probable COVID-19 infection and Infrequent real-time/written contact with family – Adjustment for and interactions with physical and mental health conditions.

Outcome	Exposure	Model	OR	p-value	CI (lower)	CI (upper)
a) Overall						
Infrequent real-time/written contact with family, COVID-19 w1	COVID-19 infection	1	0.698	0.184	0.410	1.187
	COVID-19 infection	2	0.699	0.190	0.410	1.193
Infrequent real-time/written contact with family, COVID-19 w2	COVID-19 infection	1	0.998	0.991	0.649	1.533
	COVID-19 infection	2	1.021	0.923	0.666	1.566
b) Interaction effects with mental and physical health conditions						
Infrequent real-time/written contact with family, COVID-19 w1	COVID-19 infection	3	0.277	0.190	0.041	1.889
	Mental health condition	3	0.955	0.858	0.576	1.582
	COVID-19 infection*Mental health condition	3	0.700	0.741	0.084	5.801
	New physical health condition	3	1.057	0.776	0.722	1.546
	COVID-19 infection*New physical health condition	3	0.761	0.655	0.230	2.521
Infrequent real-time/written contact with family, COVID-19 w2	COVID-19 infection	3	0.669	0.472	0.224	1.996
	Mental health condition	3	0.979	0.937	0.577	1.662
	COVID-19 infection*Mental health condition	3	2.515	0.178	0.658	9.608
	New physical health condition	3	0.720	**0.044**	0.524	0.990
	COVID-19 infection*New physical health condition	3	2.254	0.074	0.924	5.499

Note. *Sample: ELSA COVID-19 Substudy (N=5,146). Weighted pooled estimates from logistic regression models across 20 imputed datasets. Model 1: adjusted for sex, age groups, pre-COVID-19 social isolation, w2 COVID-19 infection (w2 outcome only), whether living alone, employment status, wealth, whether vulnerable to COVID-19, limiting long-standing illness; Model 2: Model 1 + mental and physical health conditions; Model 3: Model 2 + interaction effects of COVID-19 infection with sex, age, employment status, wealth, whether living alone, mental health condition, and new physical health condition. OR = odds ratio. CI = 95% confidence interval. COVID-19 infection, def 1 = Positive COVID-19 test result; or hospitalisation due to COVID-19; or at least one of the three core symptoms as defined by NHS England (i.e. high temperature, new continuous cough, and loss of sense of smell/taste). NA = no probable COVID-19 cases in the 'other not working' who reported infrequent contact with family.*

**sTable25. Associations between probable COVID-19 infection and Infrequent real-time/written contact with friends – Adjustment for and interactions with physical and mental health conditions.**						
**Outcome**	**Exposure**	**Model**	**OR**	**p-value**	**CI (lower)**	**CI (upper)**
a) Overall						
Infrequent real-time/written contact with friends, COVID-19 w1	COVID-19 infection	1	0.793	0.259	0.530	1.187
	COVID-19 infection	2	0.796	0.269	0.532	1.192
Infrequent real-time/written contact with friends, COVID-19 w2	COVID-19 infection	1	1.252	0.248	0.855	1.835
	COVID-19 infection	2	1.277	0.211	0.871	1.871
b) Interaction effects with mental and physical health conditions						
Infrequent real-time/written contact with friends, COVID-19 w1	COVID-19 infection	3	0.384	0.117	0.116	1.269
	Mental health condition	3	0.798	0.342	0.501	1.271
	COVID-19 infection*Mental health condition	3	1.954	0.242	0.636	6.000
	New physical health condition	3	0.987	0.932	0.733	1.329
	COVID-19 infection*New physical health condition	3	1.120	0.773	0.517	2.426
Infrequent real-time/written contact with friends, COVID-19 w2	COVID-19 infection	3	1.389	0.518	0.513	3.765
	Mental health condition	3	0.777	0.321	0.472	1.279
	COVID-19 infection*Mental health condition	3	1.225	0.748	0.356	4.214
	New physical health condition	3	0.874	0.338	0.663	1.152
	COVID-19 infection*New physical health condition	3	1.081	0.849	0.483	2.422
Note. *Sample: ELSA COVID-19 Substudy (N=5,146). Weighted pooled estimates from logistic regression models across 20 imputed datasets. Model 1: adjusted for sex, age groups, pre-COVID-19 social isolation, w2 COVID-19 infection (w2 outcome only), whether living alone, employment status, wealth, whether vulnerable to COVID-19, limiting long-standing illness; Model 2: Model 1 + mental and physical health conditions; Model 3: Model 2 + interaction effects of COVID-19 infection with sex, age, employment status, wealth, whether living alone, mental health condition, and new physical health condition. OR = odds ratio. CI = 95% confidence interval. COVID-19 infection, def 1 = Positive COVID-19 test result; or hospitalisation due to COVID-19; or at least one of the three core symptoms as defined by NHS England (i.e. high temperature, new continuous cough, and loss of sense of smell/taste).*						

sTable26. Associations between probable COVID-19 infection and Infrequent real-time/written contact with family and friends (total continuous score) – Adjustment for and interactions with physical and mental health conditions.						
**Outcome**	**Exposure**	**Model**	**b**	**p-value**	**CI (lower)**	**CI (upper)**
a) Overall						
Infrequent real-time/written contact with family/friends, COVID-19 w1	COVID-19 infection	1	-0.178	0.483	-0.676	0.320
	COVID-19 infection	2	-0.155	0.542	-0.653	0.343
Infrequent real-time/written contact with family/friends, COVID-19 w2	COVID-19 infection	1	0.039	0.871	-0.433	0.511
	COVID-19 infection	2	0.385	0.611	-1.099	1.869
b) Interaction effects with mental and physical health conditions						
Infrequent real-time/written contact with family/friends, COVID-19 w1	COVID-19 infection	3	0.385	0.611	-1.099	1.869
	Mental health condition	3	0.139	0.625	-0.417	0.695
	COVID-19 infection*Mental health condition	3	1.395	0.106	-0.299	3.089
	New physical health condition	3	-0.325	0.060	-0.664	0.013
	COVID-19 infection*New physical health condition	3	-0.103	0.854	-1.196	0.990
Infrequent real-time/written contact with family/friends, COVID-19 w2	COVID-19 infection	3	0.124	0.862	-1.281	1.530
	Mental health condition	3	0.193	0.472	-0.334	0.720
	COVID-19 infection*Mental health condition	3	1.177	0.151	-0.430	2.785
	New physical health condition	3	-0.279	0.088	-0.599	0.042
	COVID-19 infection*New physical health condition	3	-0.040	0.940	-1.075	0.996

Note. *Sample: ELSA COVID-19 Substudy (N=5,146). Weighted pooled estimates from linear regression models across 20 imputed datasets. Model 1: adjusted for sex, age groups, pre-COVID-19 social isolation, w2 COVID-19 infection (w2 outcome only), whether living alone, employment status, wealth, whether vulnerable to COVID-19, limiting long-standing illness; Model 2: Model 1 + mental and physical health conditions; Model 3: Model 2 + interaction effects of COVID-19 infection with sex, age, employment status, wealth, whether living alone, mental health condition, and new physical health condition. CI = 95% confidence interval. COVID-19 infection, def 1 = Positive COVID-19 test result; or hospitalisation due to COVID-19; or at least one of the three core symptoms as defined by NHS England (i.e. high temperature, new continuous cough, and loss of sense of smell/taste).*

sTable27. Relationship between pre-pandemic psychological distress and probable COVID-19 infection.					
**Outcome**	**Exposure**	**OR**	**p-value**	**CI lower**	**CI upper**
Probable COVID-19 infection (June-July 2020)	Pre-pandemic Depression (2018-19)	1.944	**0.001**	1.327	2.848
	Pre-pandemic Anxiety – ONS scale (2018-19)	1.039	0.139	0.988	1.093
	Pre-pandemic Poor Quality of Life (2018-19)	1.043	**0.000**	1.019	1.068
	Pre-pandemic Loneliness (2018-19)	1.035	0.322	0.967	1.107

Note. *Sample: ELSA COVID-19 Substudy (N=5,146); weighted pooled estimates from logistic regression models across 20 imputed datasets; separate models were fitted to test the association between each mental health outcome and probable COVID-19 infection, with adjustment for sex, age groups, whether living alone, employment status, wealth, whether vulnerable to COVID-19, and limiting long-standing illness; OR = odds ratio; CI = 95% confidence interval.*
